# A novel soft cardiac assist device based on a dielectric elastomer augmented aorta: An in vivo study

**DOI:** 10.1002/btm2.10396

**Published:** 2022-08-22

**Authors:** Thomas Martinez, Silje Ekroll Jahren, Armando Walter, Jonathan Chavanne, Francesco Clavica, Lorenzo Ferrari, Paul Philipp Heinisch, Daniela Casoni, Andreas Haeberlin, Markus M. Luedi, Dominik Obrist, Thierry Carrel, Yoan Civet, Yves Perriard

**Affiliations:** ^1^ Integrated Actuators Laboratory (LAI), École polytechnique fédérale de Lausanne (EPFL) Neuchâtel Switzerland; ^2^ ARTORG Center for Biomedical Engineering Research University of Bern Bern Switzerland; ^3^ Department of Congenital and Pediatric Heart Surgery, German Heart Center Munich Technical University of Munich Munich Germany; ^4^ Division of Congenital and Pediatric Heart Surgery University Hospital of Munich, Ludwig‐Maximilians‐University Munich Germany; ^5^ Experimental Surgery Facility University of Bern Bern Switzerland; ^6^ Department of Cardiology, Bern University Hospital Inselspital University of Bern Bern Switzerland; ^7^ Department of Anaesthesiology, Bern University Hospital Inselspital University of Bern Bern Switzerland; ^8^ Department of Cardiac Surgery University of Zurich Zurich Switzerland

**Keywords:** cardiac assist device, counterpulsation, dielectric elastomer actuator, in vivo experiment

## Abstract

Although heart transplant is the preferred solution for patients suffering from heart failures, cardiac assist devices remain key substitute therapies. Among them, aortic augmentation using dielectric elastomer actuators (DEAs) might be an alternative technological application for the future. The electrically driven actuator does not require bulky pneumatic elements (such as conventional intra‐aortic balloon pumps) and conforms tightly to the aorta thanks to the manufacturing method presented here. In this study, the proposed DEA‐based device replaces a section of the aorta and acts as a counterpulsation device. The feasibility and validation of in vivo implantation of the device into the descending aorta in a porcine model, and the level of support provided to the heart are investigated. Additionally, the influence of the activation profile and delay compared to the start of systole is studied. We demonstrate that an activation of the DEA just before the start of systole (30 ms at 100 bpm) and deactivation just after the start of diastole (0–30 ms) leads to an optimal assistance of the heart with a maximum energy provided by the DEA. The end‐diastolic and left ventricular pressures were lowered by up to 5% and 1%, respectively, compared to baseline. The early diastolic pressure was augmented in average by up to 2%.

## 1 INTRODUCTION

Congestive heart failure (CHF) is a progressive and debilitating condition affecting a substantial proportion of the elderly population. It is estimated that CHF causes more deaths than all cancer cases combined, and that approximately 26 million people worldwide suffer from the disease. A total of 5.8 million of these patients reside in the United States, with an estimated economic impact of 40 billion dollars on the country's economy in terms of medical costs and productivity loss.^1^ While drugs are used to improve heart function and relieve symptoms, they usually fail to restore cardiac function in the long term. Heart transplantation is the gold standard for patients with severely impaired left ventricular dysfunction who cannot be treated otherwise. Due to the lack of donor organs, however, cardiac assist devices for permanent hemodynamic support remain an unmet clinical need. A safe and fully implantable system, capable of restoring cardiac function and thus eliminating the need for heart transplantation, should be used to achieve this objective.^1^ Currently, existing cardiac assist devices are used as bridge to recovery, bridge to next decision, bridge to transplantation, and, more recently, as destination strategy.^2^ Among these devices, ventricular assist devices (VADs) based on rotary elements (axial and centrifugal pumps) are currently the most widely adopted solutions. Although pulsatile flow may be preferred over continuous flow, as it is closer to normal physiology and favors optimal tissue perfusion,^3^ current VADs can only ensure a constant laminar flow.^4^ Moreover, due to high shear stress on the blood cells caused by the rotating parts, they are associated with a higher risk of hemolysis and thrombosis, which forces patients to depend on permanent oral anticoagulation for the duration of the support.^5^

In this feasibility study, we therefore focus on the aortic counterpulsation (ACP) principle, which does not require anticoagulation therapy in the majority of cases^6^ and help to preserve or even enhance pulsatile flow in the aorta.

The basic principle is similar for all ACP devices: (i) they augment early diastolic pressure to increase flow in the coronary arteries and (ii) they reduce the resistance to systolic output (afterload). Depending on their location within the aorta, ACP devices are classified as: intra‐aortic,^7^, ^8^ extra‐aortic,^9^, ^10^, ^11^ and para‐aortic^12^ counterpulsators. The different characteristics of these devices are summarized in Table 1. Among all the ACP devices, the intra‐aortic balloon pump (IABP) has been the most widely used in clinical settings since decades. Its balloon is inflated in the aorta during diastole, which leads to augmentation of the diastolic pressure, while the presystolic deflation of the balloon leads to a sudden reduction of the afterload immediately before the opening of the aortic valve.^7^, ^8^ In general, IABP are used in high‐risk patients as a short‐term support to other solutions (e.g., VADs). However, patients may suffer from bleeding, hemorrhage, infections, limb and mesenteric ischemia.^6^ To overcome these significant limitations, extra‐aortic counterpulsation devices were designed by wrapping and compressing the aorta externally during diastole. In this direction, C‐pulse is an extra‐aortic balloon (EAB) counterpulsation device, pneumatically actuated, which has shown promising results^9^ in terms of improved coronary flow and ejection fraction. However, in the context of long‐term cardiac support, the pneumatic actuation of existing ACP devices is a limiting factor due to the large size of the gas sources. Large transcutaneous pneumatic drivelines (greater in size than the electric lines used for VADs) are required and can cause severe infections. Additionally, pneumatic actuation leads to long inflation times and can introduce uncontrollable delays in the device operation. Thus, a fully implantable solution for these devices is difficult to envision.

**TABLE 1:** Comparison of the different types of aortic counterpulsation devices

Type	Activation type	Displaced volume	Position	Activation speed	Obstruction of the aorta
Intra‐aortic balloon	Pneumatic	25–50 ml (28)	Inside the aorta	250 ms (29)	Yes
Extra‐aortic balloon	Pneumatic	7 ml^10^	Around the aorta	50 ms^10^	Yes
Para‐aortic balloon	Pneumatic	30 ml (30)	External to the aorta	100 ms (31)	No
Dielectric elastomer augmented aorta	Electric	<2 ml	Replacement of the aorta	<20 ms	No

*Note*: The electrical activation of the dielectric elastomer augmented aorta allows for fast activation of the device. Furthermore, the proposed design does not obstruct the blood flow in the aorta.

Dielectric elastomer actuators (DEAs) have emerged as a noteworthy alternative solution to build cardiac assist devices because they are soft devices (in contrast to rigid VADs) and only require an electric signal to be actuated. DEAs consist of a hyperelastic dielectric membrane placed between two compliant electrodes. When a voltage is applied between the electrodes a pressure known as a Maxwell pressure compresses the dielectric elastomers in the thickness direction allowing it to expand in the other directions.^13^ In our previous study,^14^ we introduced an augmented aorta based on such DEAs. When exposed to the blood pressure, the soft device passively inflates. The activation of the device allows to expand the device further and thus to control the compliance of the system. DEAs' low weight and the absence of bulky drive lines are promising advantages compared to existing technology in view of a fully implantable system and long‐term cardiac support. Moreover, as only electrical activation is needed, the activation time of the device can be much lower than for pneumatic actuators and trans‐cutaneous wireless power transfer could be achieved^15^ to reduce infections.

In this study, we tested the feasibility of the DEA augmented aorta in an acute in vivo porcine model. The DEA was implanted in the descending aorta of five pigs. The aim was to show that the DEA could provide support to the heart by lowering the end‐diastolic pressure, and augment coronary flow by increasing early aortic diastolic pressure. Additionally, the optimal activation and deactivation parameters in terms of activation duration and delay relative to aortic valve opening were investigated and reported.

## 2 RESULTS

### 2.1 The DEA acts as a counterpulsation system

**FIGURE 1:**
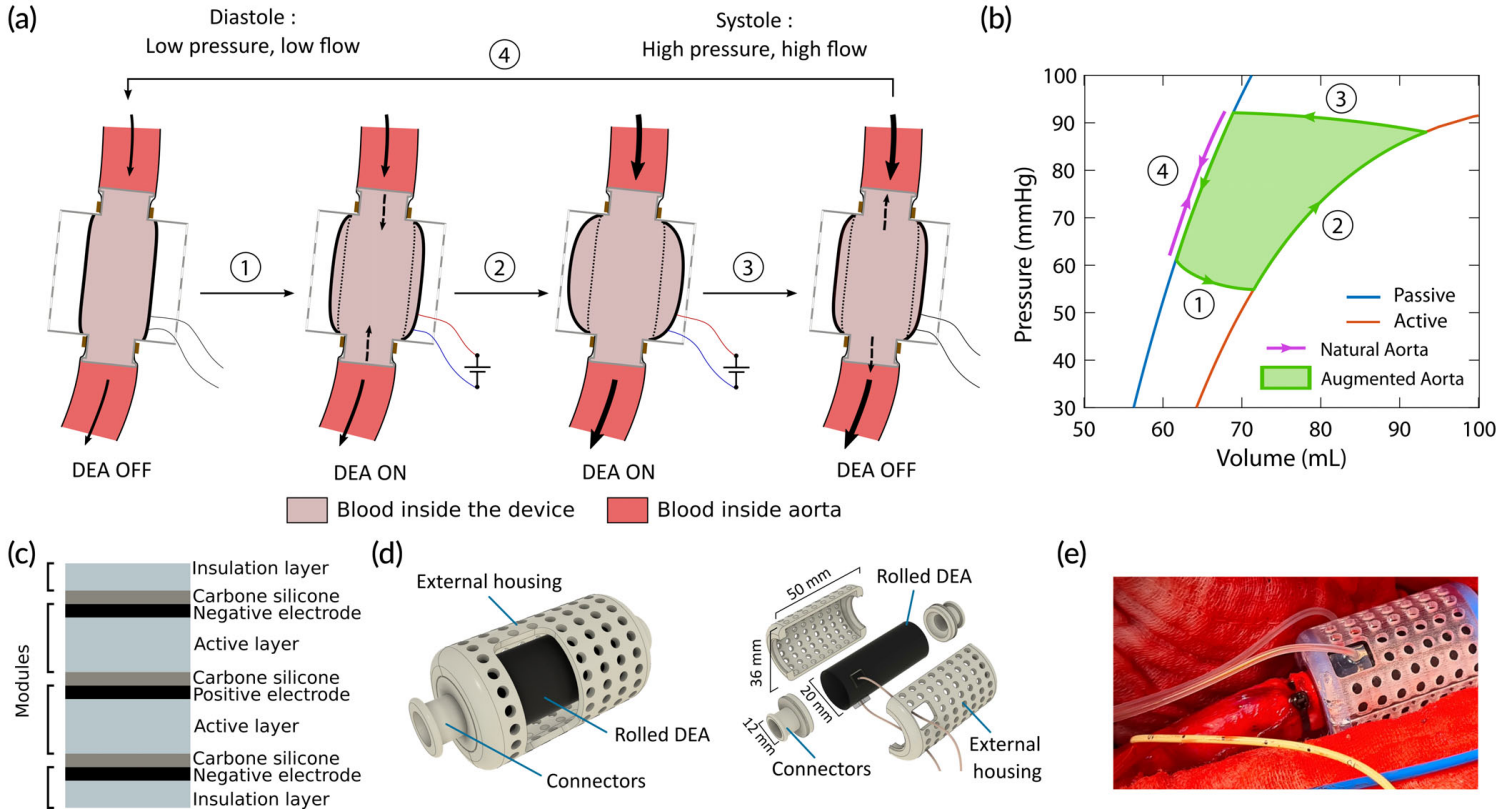
Working principle and fabrication of a multilayered DEA augmented aorta. (a) Working principle of the dielectric elastomer actuator (DEA) as an augmented aorta. (1) At the end of diastole, the DEA is activated and expands decreasing the aortic pressure. (2) The increase of aortic pressure during the systole further inflates the active DEA. (3) The deactivation of the DEA releases the stocked blood influencing the blood flow and increasing the aortic pressure when the DEA returns to its passive state. (4) The pressure decrease during diastole reduces the DEA diameter back to its initial state. (b) Pressure–volume characteristics of the DEA (passive [blue] and active [orange] state) and the natural aorta (purple). By changing the compliance of the device by activation and deactivation, the DEA can supply energy to the cardiovascular system (green area). (c) Cross‐sectional view of the multilayer stack of Elasostil films, which is rolled twice to create the tubular DEA device. One module consists of an Elastosil sheet with a compliant electrode coated at its surface. In this configuration, the positive electrode is surrounded by negative ones limiting the risks of electric breakdown with the surrounding area. (d) Assembly view and exploded view of the final device used in vivo with the rolled DEA and the 3D‐printed connectors and external housing. (e) Photographic image of the anastomosis of the device in vivo. The fixation of the device in the descending aorta is achieved with plastic clamp ties.

The DEA is a cylindrical tube that replaces a section of the descending aorta to support the heart by counterpulsation. In its passive state, the compliant nature of the DEA mimics the elastic behavior of the aorta, storing energy during systole and releasing it during diastole. Figure 1a shows the activation timing of the DEA when operating as a counterpulsation device as described in the literature.^14^ In Phase 1, the DEA is activated just before the start of the systolic phase where pressure and blood flow in the descending aorta are low, creating a small depression in the aorta and thereby increasing momentarily the flow upstream of the DEA and decreasing the flow downstream of the DEA. During systole (phase 2), the flow and pressure increase in the aorta due to left ventricular ejection leading to a further increase of the DEA radius. At the end of systole, the DEA is deactivated resulting in a drastic decrease of the radius (Phase 3) and thus an increase of the diastolic pressure. Finally, the pressure decreases during diastole (Phase 4) and the DEA is returning to the initial state. The implantation of the DEA and its working principle as a counterpulsation system are shown in the supplementary materials Video S1.

This activation scheme has two main advantages. Figure 1b shows the pressure–volume characteristics of the DEA compared to the aortic characteristics. The activation cycle of the DEA is represented in green while the deformation of the aorta only follows a line (in purple) assuming no viscoelastic loss. If not activated, the DEA acts like a natural passive aorta, storing and releasing the elastic energy without any addition of work. When activated, the DEA can provide supplementary energy to support the heart. Moreover, by actuating when the pressure is low and releasing when the pressure is high, we can maximize the energy provided by the DEA. On a physiological level, the device can store more blood and decrease aortic pressure by activating it just before systole. By releasing the additional blood volume stored in the DEA at the beginning of diastole, the pressure in the aorta increases, thereby, augmenting the coronary flow.

### 2.2 Dielectric elastomer augmented aorta

For the DEA to work as an augmented aorta, two issues must be addressed: maximizing the actuation effect of the device and ensuring good sealing of the device anastomosis with the aorta. To obtain high displacement while keeping the activation voltage as low as possible, the DEA was manufactured as a multilayered device.^16^ The DEA comprises a stack of four Elastosil films from Wacker Chemie (Figure 1c, light blue layers). The top and bottom elastomer layers are 20 μm thick and passive, acting as insulation from the surrounding environment, while the two internal elastomer layers are 100 μm thick and active. Compliant electrodes made of carbon‐doped silicone (Figure 1c, black layers) were screen‐printed on the elastomer sides with a silver ink line crossing the electrode to allow for electrical connection in the end. All the sheets were then glued together with a silicone glue lightly doped in carbon (Figure 1c, gray layers).^17^ The stack was then rolled twice and glued together with liquid silicone around a 20 mm diameter poly(methyl methacrylate) (PMMA) cylinder and cut at the desired dimensions. This configuration of active layers allowed for positive electrodes to be encapsulated between negative electrodes and ensured the electric field is confined inside the DEA.

All the electrical connections were realized by coating the extremities of the silver ink lines in carbon‐doped silicone and by adding electrical wires. Finally, a silicone casting around the electrical connections ensured the insulation with the surrounding environment and protected the pig from the high voltages up to 10 kV. For the final device used in the in vivo experiments, the DEA was inserted in a 3D‐printed housing (Figure 1d). The DEA was clamped between the two parts of the housing—the connectors and the external housing—to ensure sealing. The biggest diameter of the connector, close to the device, matched the internal radius of the DEA (Figure S3). The smallest diameter section was designed to fit into the aortic lumen. During the in vivo experiment, this section of the connector was inserted into the aorta and a plastic clamp tie was tightened around the aorta for the anastomosis (Figure 1e). The small step at this end of the connector ensured the sealing of the anastomosis. The external housing limited the radial expansion of the DEA to avoid electromechanical instability that could lead to early breakdown of the actuator.^18^ Figure 1d shows the overall dimensions of the device. Two different types of DEAs were made with 29 mm or 39 mm active electrode lengths corresponding, respectively, to 40 and 50 mm total device lengths. The overall thickness and thus the compliance of the DEA were designed to be rigid enough when passive while still deforming sufficiently when actuated. In the following, we will only refer to the DEA by their active lengths. In both cases, with the selected designs and the blood pressure levels, the DEA requires very high‐voltage levels (>6 kV) to obtain significant deformation. During the in vivo experiments, the actuation voltage was selected as trade‐off between maximum deformation and electric breakdown limit of the device.^18^ The first activation level for the measurements started at 6 kV, and it was then increased to 6.5 and 7 kV if the DEA did not break before.

### 2.3 The device is synchronized to the heart cycle in vivo

**FIGURE 2:**
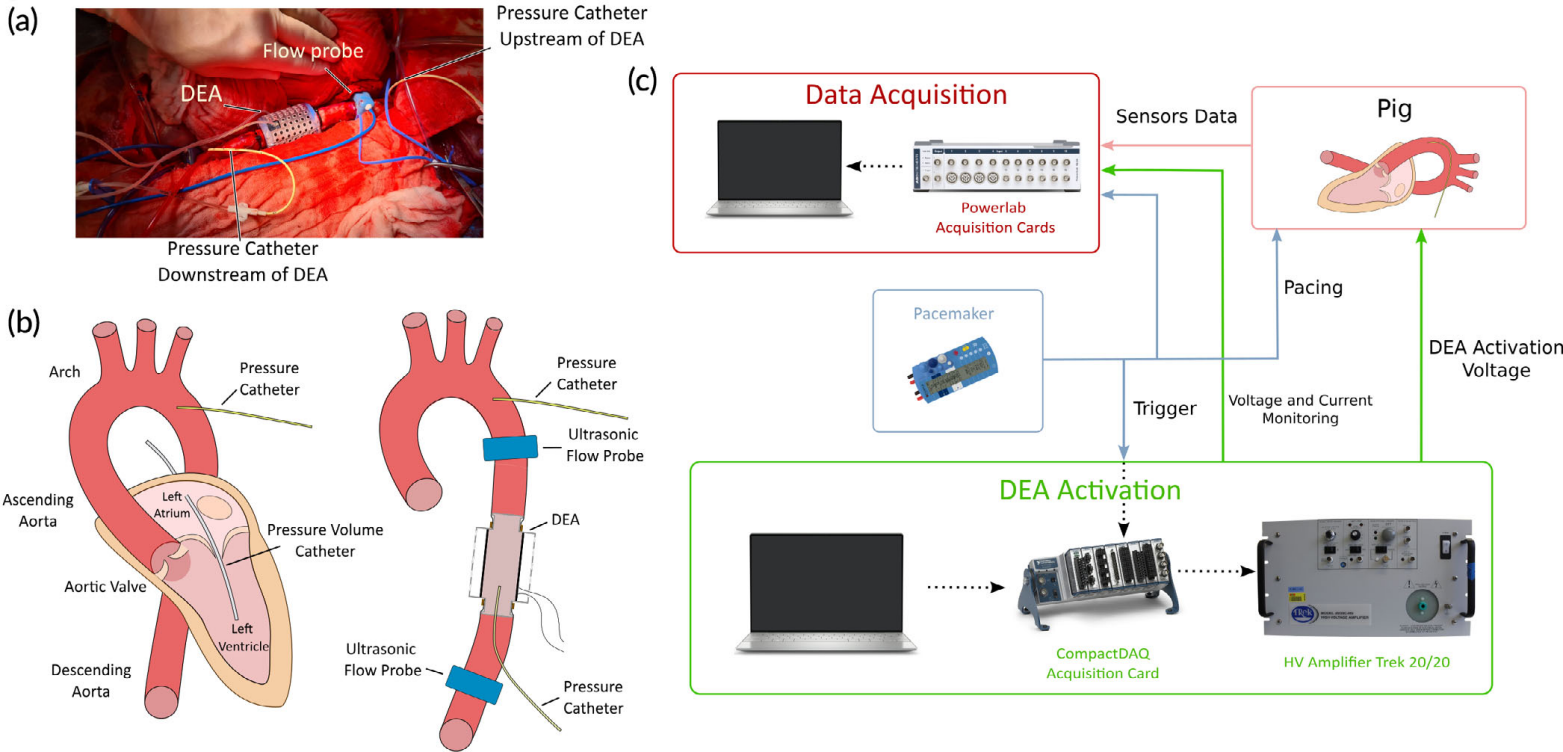
Sensors implantation and hardware actuation and acquisition scheme. (a) Photographic image of the implantation of the dielectric elastomer actuator (DEA) device in the descending aorta during the in vivo experiment. (b) Schematic of the left section of the heart showing the different sections of the aorta with the information on the position of the DEA and the different sensors used during the in vivo experiment. (c) Overview of the hardware setup used during the in vivo experiments. The DEA is activated at very high voltages in synchronization with the pacing signal from the pacemaker through the CompactDAQ and the LabVIEW software. HV, high voltage

**FIGURE 3:**
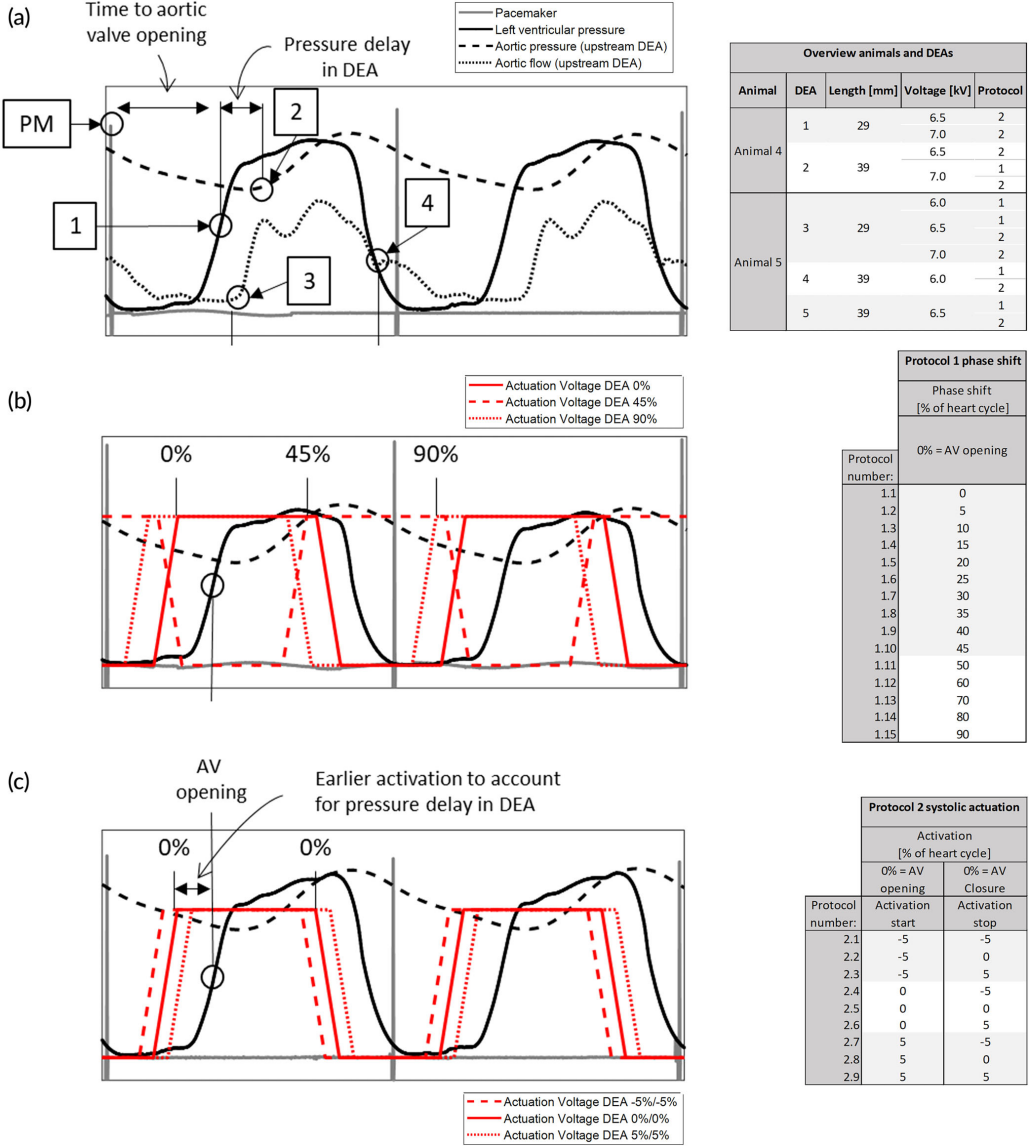
Synchronization of the dielectric elastomer actuator (DEA) actuation with the heart cycle and definitions of the tested protocols. (a) Pressure, flow, and pacemaker signals for two consecutive heart cycles. The points (1–4 and PM) used to estimate aortic valve opening and closure, as well as time delay between left ventricular pressure and the pressure upstream of the DEA are marked. PM: pacemaker signal peak, 1: mid‐point of left ventricular pressure increase as estimate for aortic valve opening, 2: delayed increase of pressure upstream of the DEA as estimate for aortic valve opening, 3: delayed increase in aortic flow upstream of DEA as estimate for aortic valve opening, and 4: delayed flow equivalent to dicrotic notch as estimate for aortic valve closure. 1‐PM: time to aortic valve opening after pacemaker signal, 2‐1: time delay of the pressure upstream of the DEA compared to the left ventricle, 4‐3: systolic duration. (b) Pressures, pacemaker signals, and three examples of actuation voltage of the experimental protocol 1, as well as a table with the full overview of the Protocol 1. In this protocol, the actuation duration was kept constant at the estimated duration of systole, and then the start of actuation was delayed for different phase shifts (in % of the heart cycle); 0% was defined as actuation start synchronized to the opening of the aortic valve (AV) (considering the pressure delay in the DEA, with earlier activation to allow the pressure wave time to reach the AV at opening). (c) Pressures, pacemaker signals, and three examples of actuation voltage of the experimental protocol 2, as well as a table with the full overview of the Protocol 2. In this protocol, the actuation was mainly during systole and start and end times of DEA actuation were delayed with respect to aortic valve opening and closure, respectively, with −5% (before), 0% (at) and 5% (after) delay in percentage of heart cycle; 0% delay corresponds to synchronization with aortic valve opening and closure for the start and end times, respectively (considering the pressure delay in the DEA, with earlier activation to allow the pressure wave time to reach the AV at opening). The two protocols were performed in all animals as seen in the overview.

**FIGURE 4:**
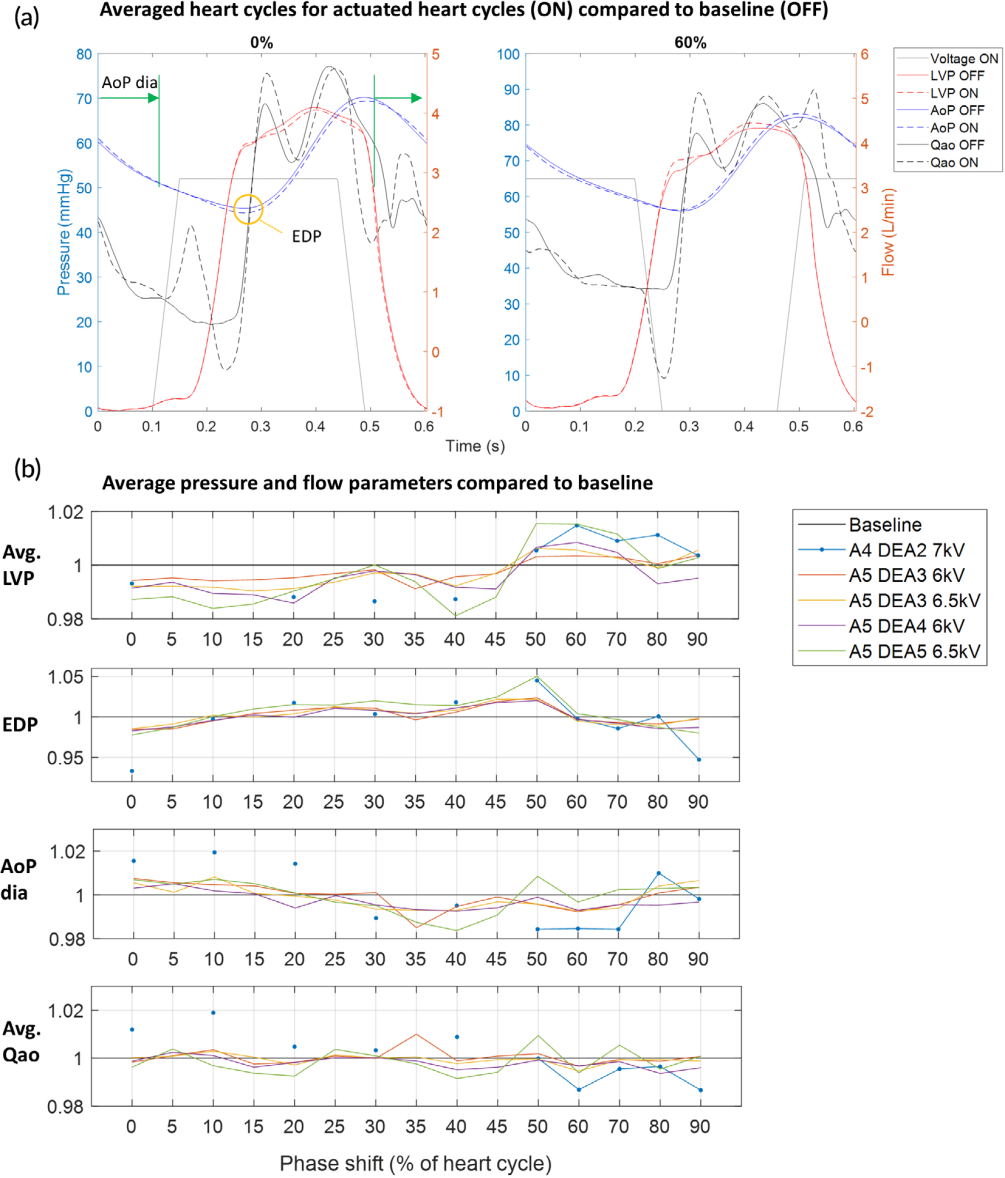
Demonstration of the assistance of the device in vivo and the influence of the activation timing on the hemodynamic parameters. (a) Averaged pressure and flow waveforms for actuated heart cycles (ON = DEA turned on) compared to baseline (OFF = DEA turned off) for two different phase shifts of Protocol 1. (b) Average values for pressure and flow parameters compared to baseline for each phase shift and each dielectric elastomer actuator (DEA) tested with Protocol 1. AoP, aortic pressure (upstream of DEA); avg., average; AoP dia, Aortic pressure early diastole; A4‐5, animal 4‐5; EDP, end‐diastolic pressure; LVP, left ventricular pressure; Qao, aortic flow (upstream of DEA)

We tested the DEA in the descending aorta in a porcine model (*n* = 5). The pigs (Edelschwein pigs, 50 kg, mixed sex) had the DEA implanted via left‐sided thoracotomy (Figure 2a), and the heart rate was controlled using a pacemaker at 100 bpm. The control and data acquisition setup in the operating room enabled recording the left ventricular pressure and volume, aortic pressures and flows upstream and downstream of the device (Figure 2b,c). The DEA was synchronized to the heart cycle using the pacemaker signal as trigger (Figure 2c) and delayed with respect to the pacemaker signal to synchronize with different time points of the heart cycle. Figure 3a shows pressure, flow, and the pacemaker signal for two consecutive heart cycles together with the different time points used for synchronization of the device to the heart cycle. Because the DEA was positioned in the descending aorta, the systolic pressure wave generated by the left ventricle (Figure 3a, point 1) reached the DEA with a time delay (Figure 3a, point 2). This time delay (approximately 80 ms in Animal 5) was estimated and removed for the actuation of the DEA (Figure S9) to ensure that the pressure waves generated by the activation and deactivation of the DEA had enough time to reach the aortic valve. To find the optimal synchronization between the heart cycle and the DEA, the DEA was tested in vivo for different actuation delays in two protocols. In Protocol 1 (Figure 3b), the actuation duration was constant and equal to the systolic duration and the start of actuation was phase shifted (delayed) compared to the pacemaker signal for 15 different phase shifts in percentage of the heart cycle (all phase shifts are listed in the table of Figure 3b, 0% corresponds to start of actuation synchronized with aortic valve opening). In Protocol 2 (Figure 3c), the expected best synchronization of the DEA with actuation mainly during systole was tested for different activation start and end times. The start and end times were tested before (−5% of heart cycle), at (0% of heart cycle) and after (5% of heart cycle) aortic valve opening and closure, respectively, leading to a variable actuation duration (all combinations are listed in the table of Figure 3c). Before and after each device actuation period (for both protocols) the device was turned off, and these heart cycles were used as baseline for each actuation period. Figure 3b shows three examples of actuation phase shifts of Protocol 1, and Figure 3c shows three examples of different DEA activation start and end times of Protocol 2. The actuation of the 0% cases started earlier than actual aortic valve opening, due to the time delay discussed above and ended earlier than actual closure. This time delay has been considered in all reported protocols of this manuscript, meaning that the DEA was always activated this time delay earlier than the wanted synchronization to the heart. The table in Figure 3a gives an overview of the two animals used for the analysis and of the DEAs tested with the corresponding protocols, actuation voltages, and DEA lengths. Different problems (e.g., early device breakdown) occurred in the three first animals (see Methods and Materials and Table S1 in the Supplementary Materials for further details). Several DEAs were tested in each animal. The following parameters were calculated for all analyzed heart cycles (20 consecutive baseline cycles and 20 consecutive actuated cycles): average left ventricular pressure (avg. LVP), end‐diastolic pressure (EDP), early diastolic aortic pressure (AoP dia, calculated as the average aortic pressure after valve closure until 0.1 s before aortic valve opening to avoid end‐diastole), and average aortic flow (avg. Qao). Additionally, averaged pressure and flow waveforms were calculated. The left‐ventricle volume measurement displayed chaotic behavior independent of the activation (Figure S12) and was not used in the analysis. Figure 4a shows the averaged heart cycles, and how the parameters EDP and AoP dia are defined.

### 2.4 Targeting the best actuation scheme

The actuation of the DEA for different phase shifts (Protocol 1) changed the pressures and flow in the aorta differently compared to baseline. Figure 4a shows the averaged pressure and flow heart cycles for two different phase shifts compared to baseline. The aortic pressure (upstream of the DEA) is shifted up or down during the different phases of the heart cycle depending on the phase shift of the actuation, while the left ventricular pressure changed only during systole because aortic valve is closed during diastole (Figure 4a). The aortic flow upstream of the DEA reacts to the device actuation phase shift: a clear wave is seen just after start of activation and just after end of activation for all phase shifts, compared to baseline. The increase in flow just after starting the activation is caused by the decompression wave generated when the DEA expands and thus sucks blood into the device. The decrease in flow just after the end of activation is caused by the compression wave generated when the DEA deflates back to its passive state and pushes out blood. Figure 4b shows the average pressure and flow parameters for each phase shift and for each DEA tested compared to baseline. Despite some differences in the lengths of the DEAs and the activation voltage, we can observe similar evolution of the pressure parameters over the range of phase shifts (this evolution is further detailed in Figure S10A where all tests are aggregated). Table 2a gives an overview of the average values and standard deviation of the pressure and flow parameters for Protocol 1 compared to baseline with all tested DEAs and voltages aggregated (Table S2A gives an overview of the average, maximum and minimum values and standard deviations of all pressure and flow parameters). The nonsignificant changes, compared to baseline, are marked (*p > 0.05). For phase shifts where the actuation is in the second half of diastole or overlaps with systole (60%–10%), the EDP decreased of 0.2%–2.7% compared to baseline (Figure 4b and Table 2a, *p* < 0.05), and for all other phase shifts the EDP increased. A lower EDP means that the pressure in the aorta was decreased, making it easier for the left ventricle to eject blood (reduced afterload). However, if the DEA actuation was ended during end‐diastole or first part of systole (50%–70%) the compression wave generated by the deactivation of the DEA increased the LVP during systole, while an activation during systole (0%–45%) decreased the LVP during systole. These two effects can be seen in the average LVP which was lowered by 0.4%–1.2% compared to baseline for the phase shifts 0%–45% (Figure 4b and Table 2a, *p* < 0.05), while it was increased (50%–70%) or constant (80%–90%) for all other phase shifts.

**TABLE 2:** Variation of the pressure and flow parameters in vivo due to the influence of the DEA augmented aorta

A	Protocol 1: Actuation phase shift														
Phase shift (%)	0	5	10	15	20	25	30	35	40	45	50	60	70	80	90
avg. LVP	−0.8 ± 1.8	−0.8 ± 1.7	−1.2 ± 1.9	−1.0 ± 1.7	−1.0 ± 1.9	−0.5 ± 1.7	−0.4 ± 1.9	−0.5 ± 1.7	−1.0 ± 1.9	−0.7 ± 1.7	0.7 ± 1.9	1.0 ± 1.8	0.6 ± 1.8	0.1 ± 1.9^a^	0.2 ± 1.7^a^
EDP	−2.7 ± 2.1	−1.2 ± 0.7	−0.2 ± 0.7	0.4 ± 0.8	0.9 ± 1.0	1.3 ± 0.8	1.0 ± 1.0	0.5 ± 1.0	1.1 ± 0.9	2.1 ± 0.9	3.2 ± 1.5	−0.2 ± 0.7	−0.8 ± 0.7	−0.9 ± 0.9	−1.8 ± 2.0
AoP dia	0.8 ± 0.9	0.4 ± 0.8	0.8 ± 1.0	0.3 ± 0.8	0.2 ± 1.1^a^	−0.1 ± 0.8	−0.5 ± 0.9	−1.0 ± 0.9	−0.8 ± 0.9	−0.5 ± 1.0	−0.3 ± 1.2	−0.8 ± 0.9	−0.6 ± 1.0	0.3 ± 0.9	0.2 ± 0.9
avg. Qao	0.1 ± 1.7^a^	0.2 ± 1.4	0.5 ± 1.9	−0.3 ± 1.4	−0.2 ± 1.7	0.2 ± 1.6^a^	0.1 ± 1.6^a^	0.2 ± 1.6^a^	−0.2 ± 1.9	−0.2 ± 1.5^a^	0.2 ± 1.7^a^	−0.6 ± 1.6	0.0 ± 1.6^a^	−0.3 ± 1.5	−0.3 ± 1.6

B	Protocol 2: Different activation start and end point with actuation during systole								
Activation (ON, OFF) (%)	(−5, −5)	(−5, 0)	(−5, 5)	(0, −5)	(0, 0)	(0, 5)	(5, −5)	(5, 0)	(5, 5)
avg. LVP	−0.8 ± 1.6	−0.8 ± 1.6	−0.4 ± 1.6	−0.9 ± 1.6	−0.8 ± 1.6	−0.7 ± 1.6	−1.0 ± 1.5	−1.0 ± 1.6	−0.8 ± 1.6
EDP	−3.0 ± 1.8	−2.3 ± 1.8	−2.0 ± 1.8	−3.3 ± 1.2	−2.9 ± 1.5	−2.6 ± 1.5	−2.2 ± 0.9	−1.9 ± 0.9	−1.6 ± 1.0
AoP dia	0.5 ± 0.9	1.2 ± 0.9	1.4 ± 0.9	0.5 ± 1.1	0.9 ± 1.0	1.1 ± 1.0	0.4 ± 0.9	1.0 ± 1.0	1.1 ± 1.0
avg. Qao	−0.2 ± 1.6^a^	0.1 ± 1.8^a^	0.4 ± 1.7	−0.0 ± 1.7^a^	0.1 ± 1.7^a^	0.2 ± 1.7^a^	0.1 ± 1.8^a^	0.1 ± 1.8^a^	0.4 ± 1.8^a^

*Note*: The results of all DEAs and voltages tested for Protocol 1 (A) and Protocol 2 (B) are combined to evaluate the influence of the DEA on the pressure and flow parameters. The numbers are given in percentage change (%) of device actuated cycles compared to baseline.

Abbreviations: AoP dia, aortic pressure early diastole; Avg., average; EDP, end‐diastolic pressure; LVP, left ventricular pressure; Qao: aortic flow.

^a^ Standard deviation is computed and nonsignificant change.

The device also affected the early diastolic aortic pressure (AoP dia, Figure 4b) depending on the phase shift. An augmentation of the early diastolic pressure of 0.2%–2% compared to baseline was seen for phase shifts with the end of actuation during late systole or early diastole (80%–15%, *p* < 0.05). An augmented aortic pressure during early diastole is expected to increase the coronary flow. For the average aortic flow (avg. Qao), it can be seen in Figure 4b that there is a small increase or decrease of the aortic flow for some cases (e.g., 15% and 40% phase shift). However, when considering the results for all the DEAs there were large variations. For 5%–10%, there was a small significant increase in flow compared to baseline (Table 2a, *p* < 0.05), and for 15%–20%, 40%, 60%, and 80%–90% (Table 2a, *p* < 0.05), there was a small significant decrease in flow. The latter phase shifts (60%–90%) correspond to an opposite effect compared to counterpulsation as it lowered the early diastolic pressure (AoP dia) and increased the average LVP and thereby loading instead of unloading the ventricle. Based on these results, the main actuation during systole (90%–10%, counterpulsation) seems like the most effective phase shifts to augment the aortic pressure during early diastole and lower the EDP as well as the average LVP. For these phase shifts, the DEA was activated during late diastole (lowering EDP) or early systole, stayed active during large parts of systole (lowering aortic pressure and average LVP), and was deactivated during late systole or early diastole (augmenting early diastolic aortic pressure). The largest effect of assistance was observed in animal 4, DEA 2 that was actuated at 7 kV. Here, the EDP was lowered on average by 5%–7% (at 90%–0% phase shift), the LVP was lowered by 1%–2% (at 0%–10% phase shift), and the early diastolic pressure was increased by 1%–2% (at 0%–10% phase shift). Figure S11 shows the raw pressure and flow signals recorded in animal 4, DEA 2 and the clear impact of the DEA actuation.

### 2.5 Interplay between the device and the cardiovascular system

**FIGURE 5:**
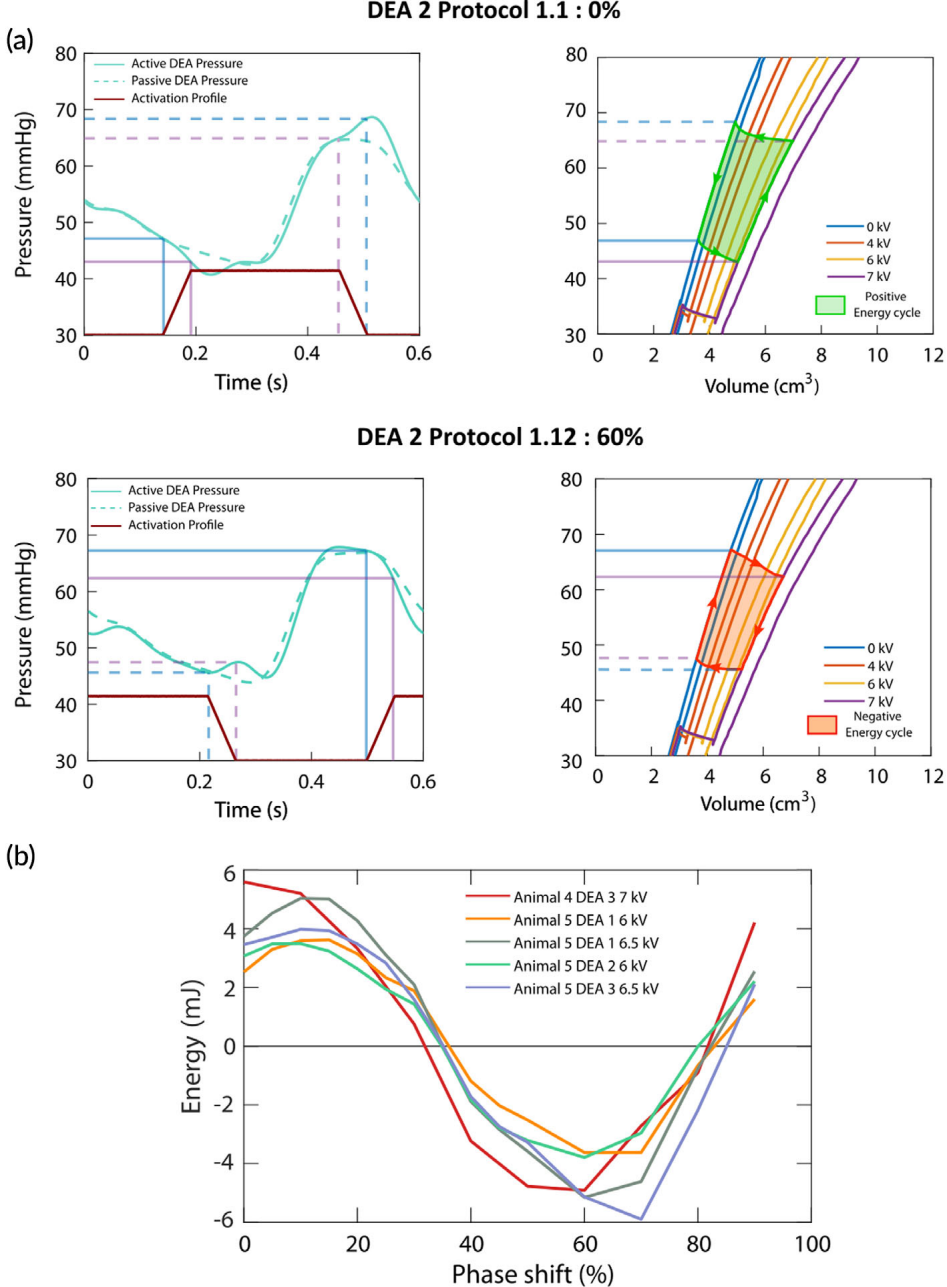
In vitro estimation of the energy supplied by the dielectric elastomer actuator (DEA) in vivo. (a) Pressure levels in the DEA during in vivo experiment and the pressure–volume characteristics with the corresponding energy cycle for 0% and 60% phase shift for DEA 2 (39 mm) at 7 kV. The light blue and purple lines represent the pressure levels in the DEA at start and end of activation and deactivation, respectively. The pressure–volume characteristics from 0 to 7 kV were obtained through in vitro measurements prior to the in vivo experiments. Depending on the activation time, the DEA will provide to (green counterclockwise energy cycle) or take (orange clockwise energy cycle) energy from the cardiovascular system. (b) Evolution of the estimated energy provided by the DEA as a function of the shift of activation for all DEAs tested for Protocol 1. The maximum energy is reached for 90%–20% phase shift.

In addition to the evolution of physiological parameters, we evaluated the influence of the DEA by estimating the energy it provided to the cardiovascular system. Figure 1b shows the pressure–volume relationship of the DEA and the natural aorta. The area of the pressure–volume cycle (green area) represents the energy provided by the DEA. However, because the DEA was enclosed within the external housing, optical access to the DEA was limited during the in vivo experiments, and the DEA tube deformation and associated volume could not be measured. To address this issue, prior characterization of the DEA was conducted in vitro in the laboratory. The aim of the characterization was to obtain the pressure–volume characteristics of the device at different activation voltages. The complete experiment is detailed in the Supplementary Materials and Figure S4B shows the picture of the setup. Figure 5a shows the pressure–volume characteristics of 39 mm DEAs for voltages up to 7 kV (Figure S5 shows the same for 29 mm DEAs). Due to the viscoelasticity of the elastomer, the characteristics present a slight hysteresis that was not considered for the energy estimation. From the in vivo experiments, for each protocol, we determined the pressure level in the DEA at activation and deactivation (Figure 5a). Then, we used these values to find the corresponding volume values from the in vitro pressure–volume characterization to have an estimation of the volume of the DEA at activation and deactivation. Finally, by computing the area of the resulting pressure–volume cycles, we obtained an estimation of the energy provided by the DEA during the in vivo experiment. Accounting for dynamic behavior, the deformation of the DEA for same pressure and activation voltage might slightly vary between the two experiments. The results show that if the DEA was activated when the aortic pressure was low (typically at the end of diastole) and deactivated when the pressure was high (end of systole, early diastole) (e.g., 0% phase shift) the energy provided by the DEA was maximized. For this case, the pressure would increase while the DEA was active leading to larger expansion, and the DEA would release and provide a larger amount of stored energy to the cardiovascular system when deactivated (Figure 5a, green area). This behavior corresponds to a counter‐clockwise pressure–volume cycle. However, if the DEA activations started at a high pressure and ended at a low pressure (e.g., 60% phase shift) the DEA was not providing energy but used energy from the cardiovascular system. The pressure would decrease during actuation reducing the expansion of the DEA (lowering the amount of stored energy) and the DEA would work against the heart when deactivated just before the start of systole (increasing EDP) and thereby be detrimental to the assistance (Figure 5a, orange area). This behavior corresponds to clockwise pressure–volume cycle. In Figure 5b, we see the estimations of the energy provided by the different DEAs for the different phase shifts during Protocol 1. For phase shifts between 90% and 40%, the DEA provided energy to the cardiovascular system with maximum values for 90%–20% phase shifts. These results confirm the timing of activation to maximize the effect of the actuator presented in the previous section.

### 2.6 Efficient counterpulsating DEA‐augmented aorta

**FIGURE 6:**
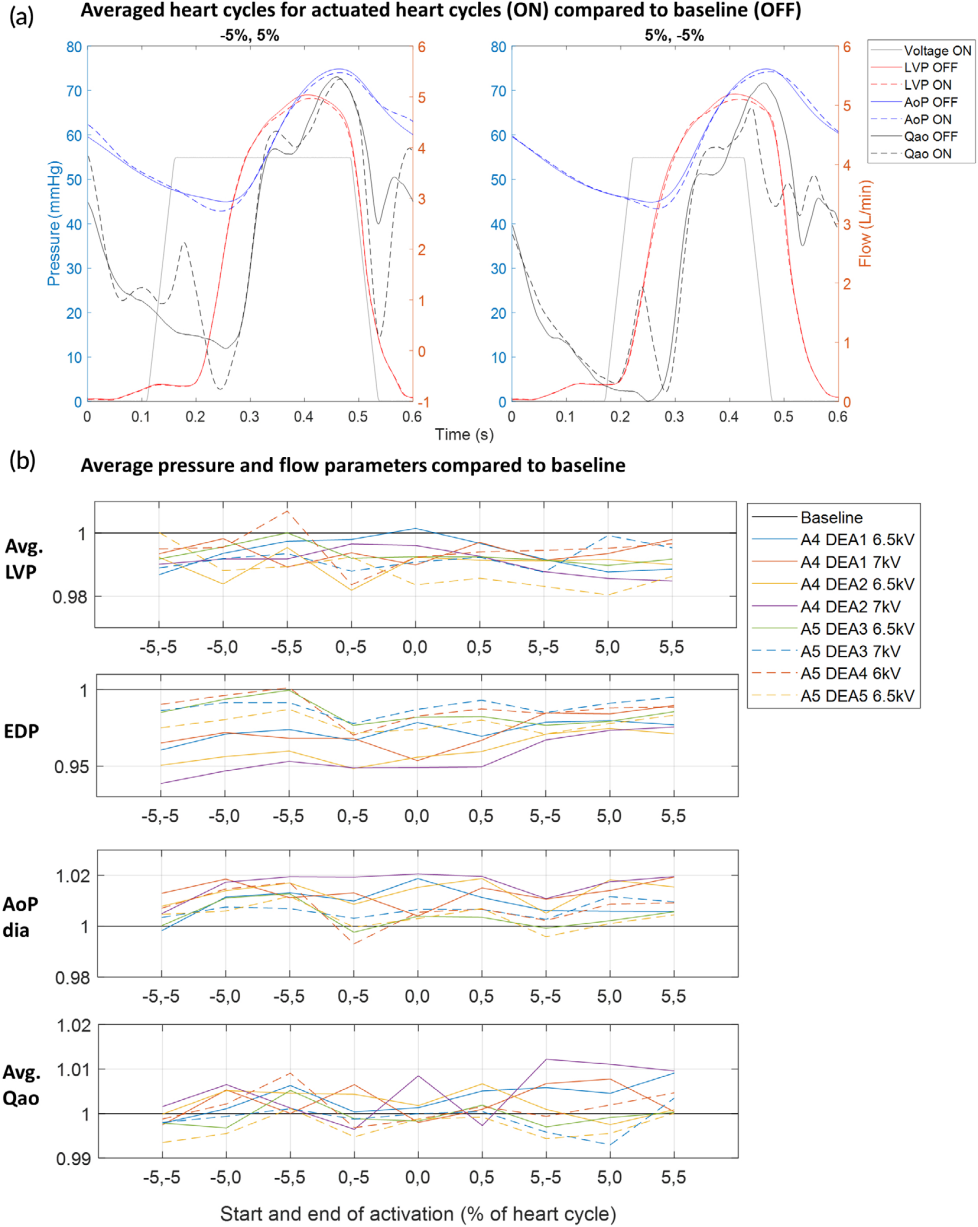
Fine tuning of the activation profile for efficient in vivo assistance and the influence of the start and end times of activation on the hemodynamic parameters. (a) Averaged pressure and flow waveforms for actuated heart cycles (ON: DEA turned ON) compared to baseline (OFF: DEA turned off) for two different activation start and end points of Protocol 2. (b) Average values for pressure and flow parameters for each dielectric elastomer actuator (DEA) tested with Protocol 2. AoP, aortic pressure (upstream of DEA); avg., average; AoP dia, aortic pressure early diastole; A4‐5, Animal 4‐5; EDP, end‐diastolic pressure; LVP, left ventricular pressure; Qao, aortic flow (upstream of DEA)

In Protocol 2, the DEA was mainly actuated during systole (corresponding to the phase shifts 90%–10%, counterpulsation principle). The best start and end times of activation, and thereby the duration of actuation, were investigated by testing three different start and end times (before [−5%], at [0%] or after [5%] aortic valve opening and closure, respectively). Figure 6a shows the averaged pressure and flow waveforms for two different start and end times of activation compared to baseline. The impact of the different start and end times were slightly different due to the timing of activation. Figure 6b shows the average values of the parameters for each DEA (this evolution is further detailed in Figure S10B where all tests are aggregated). For the pressure parameters, all DEAs showed the same trend in line with the expected behavior seen in the results of Protocol 1. However, there were large differences in the amplitude of the effect, because the devices' length, voltages, and the physiological characteristics of the animal were overall different. Table 2b gives an overview of the average values and standard deviation of all the pressure and flow parameters for Protocol 2 (Table S2B gives an overview of the average, maximum and minimum values, and standard deviations of all pressure and flow parameters). The EDP was lowered for all different start and end times (Table 2b, *p* < 0.05), but less if activation start was after aortic valve opening ([5, −5], [5, 0] and [5]). The early diastolic aortic pressure was augmented for all different start and end times (Table 2b, *p* < 0.05). However, it was more augmented if the device activation was ended at valve closure or after valve closure ([−5, 0 or 5], [0, 0 or 5], and [5, 0 or 5]), and additionally if the activation start was before valve opening (−5, 0 or 5), resulting in an activation length slightly longer than the systolic length. The average LVP decreased for all start and end times (*p* < 0.05). The mean aortic flow did not change for all start and end times (Table 2b, *p* > 0.05) except for a start time before and an end time after aortic valve opening and closure (−5, 5), respectively, where the flow was slightly increased (Table 2b, *p* < 0.05). The largest effect of assistance was observed in Animal 4, DEA 2, which was actuated at 7 kV. Here, the EDP was lowered in average with 5%, the LVP was lowered with 1%, and the early diastolic pressure was increased with 2% (at [−5, 0 or 5]).

## 3 DISCUSSION

We successfully established a soft cardiac assist device based on a DEA, which replaces a segment of the aorta and works as an augmented aorta. The DEA was implanted and tested in vivo in the descending aorta of an acute porcine model. The developed manufacturing process allowed us to create the DEA as a multilayer rolled tube with an internal diameter comparable to the porcine aortic diameter. The tube was combined with a 3D‐printed external housing, which ensured safe operation regarding electromechanical instabilities. Specific connectors have also been designed for the tight anastomosis between aorta and device.

When activated the DEA increases its internal diameter and thereby creates a decompression wave decreasing the aortic pressure. When deactivated, it returns to its passive state creating a compression wave increasing the aortic pressure. Existing counterpulsation devices (e.g., IABPs or EAB^10^) obstruct the aortic lumen when activated and this restricts their activation to the diastolic phase only (because if a balloon is inflated during systole, it would increase the afterload due to obstruction) as shown in Table 1. On the contrary, our DEA device allows cardiac assistance without aortic obstruction because the DEA's passive internal diameter is comparable to the aortic diameter. One of the major differences compared to IABP is that, while the classical IABP lies within the aorta, the DEA replaces a short segment of the aorta. In this context, the principle is similar to the para‐aortic counterpulsation device, also called para‐aortic blood pump (PABP) as both devices replace a segment of the aorta and increase its volume,^12^ allowing more freedom in terms of activation and deactivation phases. However, PABP requires a bulky pneumatic chamber hardly implantable in the thoracic cavity, and it can modify greatly the flow of blood from its natural path. Our DEA only needs an electric activation to assist the heart and does not require bulky pneumatic elements as other counterpulsation devices do. Similar attempts were conducted by Hashem et al.^19^ and Starck et al.^20^ who developed extra‐aortic counter‐pulsation devices based on: (i) shape memory alloy fibers, which contracts following Joule heating, and (ii) a ferromagnetic silicone that contracts triggered by an external magnetic field, respectively. However, the low compatibility of the working principle with human implantation and the effectiveness of these latter devices need further investigations^21^ before in vivo implementation, especially for blood pressures higher than 50 mmHg.^20^

In this in vivo study, the nonobstructive nature of the DEA made it possible to investigate the effect of actuation at different time points during the heart cycle (phase shifts). Depending on the actuation phase shift, the level of assistance was changing: with actuation at 90%–10% (counterpulsation) of the heart cycle (0% = actuation‐start synchronized to aortic valve opening) the estimated energy provided by the DEA was maximized (Figure 5b) and thereby also the unloading of the cardiovascular system. With actuation start at 60% phase shift (co‐pulsation), the estimated energy was minimized and the DEA actually increased the load on the cardiovascular system. Thus, by controlling the phase shift of actuation, it is possible to control the loading of the heart. Gradual loading of the left ventricle might be a method to stimulate recovery of patients with heart failure.^22^ Looking at the measured pressure and flow parameters, the phase shifts 90%–10% seem to be the best to lower EDP and augment the early diastolic aortic pressure (Figure 4b). To optimize the DEA assistance further, different start and end times of activation with the DEA mainly active during systole (comparable to 90%–10% phase shifts) were investigated. A start of activation just before the aortic valve opens (−5% of heart cycle [0% is defined as aortic valve opening]) and an end of activation at or just after aortic valve closure (0% or 5% of heart cycle [0% is defined as aortic valve closure]) were found to be optimal (Figure 6b). In this way, the DEA is activated at low pressure at the end of diastole and thereby decreases the EDP. It is active during systole and thereby lowers the average LVP. It is deactivated at high pressure at start of diastole and thereby increases the early diastolic aortic pressure. Hence, the optimal activation of the DEA helped reducing the left ventricle pressure during systole and the end‐diastolic aortic pressure by up to 1% and 5%, respectively, and augmenting the pressure in the aorta during diastole by up to 2%, which might enhance the coronary flow (see Table S2 for maximum, minimum, and average values for all measured hemodynamic variables).

As comparison, using IABP in patients, Kolywa et al.^23^ reported a reduction in the end‐diastolic pressure of 13.7% (from 50.9 to 43.9 mmHg) and a reduction in the average LVP of 10.8% (from 42.3 to 37.7 mmHg). The volume of the IABP balloon varied between 34, 40, or 50 cm^3^ (according to patient height). In acute porcine models using 40 cm^3^ IABP balloons, Lu et al.^12^ reported an increase in the peak diastolic aortic pressure of 26.7% (from 56 to 71 mmHg) and a reduction in the end‐diastolic pressure and in the peak systolic left ventricular pressure of 13.9% (from 43 to 37 mmHg) and 7.8% (from 76 to 70 mmHg), respectively. In the same experiments, Lu et al. used 40 cm^3^ para‐aortic devices mounted on descending aorta, which led to an increase in the peak diastolic aortic pressure of 39.2% (from 56 to 78 mmHg) and a reduction in the end‐diastolic pressure and in the peak systolic left ventricular pressure of 34.7% (from 46 to 30 mmHg) and of 27.5% (from 80 to 58 mmHg), respectively. However, in our case, the DEA volume change was much lower (<2 cm^3^) and the energy provided by the device was quite low (<7 mJ, <2% of the estimated LV energy).

Nevertheless, we were able to show that the DEA can assist the cardiac system in two animals despite these low values. The devices were originally designed for implantation in humans and therefore to work at higher pressures (between 80 and 120 mmHg). At higher pressures, the displacement would have been bigger and thus also the energy provided by the DEA and its impact on the cardiovascular system. In this study, we were limited by the pressure levels in the pigs and the breakdown voltage of the DEA and were not able to reach voltages higher than 7 kV. But we can already see the impact of supplying higher energies. Indeed, we see in Figure 4b that the greatest changes of EDP and early diastolic aortic pressure were obtained for a 39 mm DEA at 7 kV thus with more volume deformation than other DEA. We see similar trends in Figure 6b where the higher decrease of the end diastolic pressure is obtained for the same DEA at 7 kV. Nonetheless, the influence of the DEA and its energy is also linked to the specific blood pressure levels (which can vary during the in vivo experiments) and can explain the variations for similar size DEAs tested at identical voltages.

The results presented in this study serve as a proof of concept for the use of DEAs as cardiac assist device. However, some aspects still need to be further optimized before considering a potential clinical application. First, an increase of performance is required to justify the implantation of the device in a patient. The first step in this process is an optimization of the design to conform to the patient's pressure levels. By doing this, we expect to obtain higher deformation of the DEA and thus more provided energy while also increasing the stability of the device. Furthermore, we could anticipate that further in vivo experiments should focus on DEA implantation in the ascending aorta. The benefits of counterpulsation at the level of the ascending aorta are expected to be more important compared to counterpulsation in descending aorta for several reasons^24^: (i) it ensures a better synchronization to the cardiac cycle due to its proximity to the aortic valve, (ii) counterpulsation efficiency and blood volume displacement are maximized because the pulse propagation in other arteries is minimized, and (iii) the increase in coronary blood flow is also maximized,^25^, ^26^ more than with IABP. In the current study, we tested our device in anesthetized, but healthy porcine hearts. The aim of further in vivo experiments will be to include hearts with reduced systolic function such as myocardiac infection or ischemia reperfusion.

From the engineering aspect of the device before it could be considered for chronic in vivo experiments, work is already performed concerning the synchronization of the activation of the DEA with the R‐wave from an ECG instead of a pacemaker. Moreover, a full integration of the wireless high voltage power supply with transcutaneous power transfer and control of activation must be developed and tested. Finally, the DEA should have no contact with the blood, and the development of a DEA wrapped around the aorta will be crucial to make the operation less dangerous and ensures long‐term operation of the device. In the end, further studies will need to be done on the long‐term chronic implantation and impact of the device. The fatigue life of dielectric elastomers needs to be studied and their stability of operation over more than 400 million activations (10 years continuous operation). Nonetheless, we demonstrated that a DEA is a step forward toward a soft cardiac assist device more suitable compared to existing solution. The risk to create hemolysis and shear‐induced thrombocyte activation (blood clotting), major problems encountered with other cardiac devices, are minimized by augmenting the natural role of the aorta deformation to bring energy to the flow.

## 4 MATERIALS AND METHODS

### 4.1 Study design

The aim of this study was to show that our soft DEA could provide some support to the heart by lowering the end‐diastolic pressure, and augment coronary flow by increasing early aortic diastolic pressure. An acute porcine model, atrium‐paced at 100 bpm, was used to test our device in vivo in *n* = 5 pigs. The device was implanted in the descending aorta. Cardiac parameters were recorded during baseline (60 heart cycles before and 60 heart cycles after device‐actuated period) and during actuation of the device (60 heart cycles). Forty consecutive heart cycles, 20 baseline and 20 device‐actuated, for each device protocol were analyzed using MATLAB (MathWorks, Natick, USA).

### 4.2 Animals and surgical procedure

This experiment was approved by the Commission of Animal Experimentation of the Canton of Bern, Switzerland (Approval number BE14/2021). A DEA assist device was implanted in *n* = 5 Edelschwein pigs of about 50 kg (three females, two males). Anesthetic management is described in the supplementary materials S1. Left‐sided thoracotomy was performed and the pericardium was opened. After heparin administration, a left heart bypass was established via cannulation of the left atria atrium (angled tip 28 Fr. Cannula) and the descending aorta (14 Fr. Arterial Cannula) at the diaphragmic level. After initiation of left heart bypass, the descending aorta was clamped distally to the right subclavian artery and just proximal at the diaphragmic level, and a part of it excised. The device was anastomosed to the descending aorta at the place of the excised part. After implantation, the aortic clamps were removed. Temporary pacemaker leads were attached to the right atrium and a fixed rate of 100 bpm was started. The schematic of the left structure of the heart and aorta as well as the placement of the sensors is shown in Figure 2a. A pressure–volume catheter (Millar, ADInstruments, Houston, USA) was introduced into the left ventricle through the left atrium. Two water‐filled pressure catheters (Xtrans, CODAN Pvb Critical Care GmbH, Forstinning, Germany) were inserted in the descending aorta: one placed upstream of the device, toward the ascending aorta, and the other inside the DEA device. Two ultrasonic flow probes (COnfidence, Transonic Systems, Inc., Ithaca, NY, USA) were placed around the descending aorta: one upstream and the other downstream of the device. All data were logged continuously in real time using the LabChart data logger (ADInstruments). To remove all sensor hardware and software delays, and have all sensors synchronized, we performed a synchronization measurement before each experiment. Figure S8 shows the experimental setup for this measurement. All sensors (flow probes, pressure sensors, and the pressure–volume catheter) were attached at the same height to a water column. A piezo element (direct signal, no delay) attached at the same height was used as reference. A sharp increase in pressure and flow was generated by dropping a cylinder into the water column. The delay of this sharp signal of the different sensors was compared with the piezo reference and removed to synchronize all sensors. Additionally, the pressure sensors were calibrated using the same water column, measuring three reference heights of water.

### 4.3 Device synchronization and study protocol

The DEA assist device was activated and deactivated using a high‐voltage unit, which was synchronized to the pacemaker of the animal using the pacing signal as trigger. To synchronize the DEA activation to the aortic valve opening and deactivation to the aortic valve closure a few heart cycles were measured. Figure 3a shows the different points used to synchronize the activation and deactivation of the DEA to the aortic valve opening and closure, respectively. For aortic valve opening, the time between the mid‐point of the left ventricular pressure increase (Figure 3a, point 1) and the pacing signal peak (Figure 3a, point PM) was used. Because the DEA was positioned in the descending aorta, the pressure in the DEA was delayed compared to the pressure in the left ventricle. This delay was measured as the time between point 1 and the start of pressure increase in the DEA (Figure 3a, point 2) and removed for the device activation. Hence, considering this delay, the device was actuated earlier (Figure S9). The systolic length was measured as the time between flow increase (Figure 3a, point 3) and the flow equivalent to the dicrotic notch (Figure 3a, point 4) seen in the aortic flow signal and used for the length of device actuation. For all these time points, the average over several heart cycles compared to the pacemaker signal were used and they were assumed to stay constant for all heart beats in one animal during pacing at a fixed heartrate. The device was actuated with two different actuation protocols. In the first protocol (Protocol 1) the actuation length was fixed, and the start of actuation was phase shifted compared to the pacemaker signal. The phase shift was defined as the time between the aortic valve opening and the start of actuation in percentage, with 0% meaning synchronized to the aortic valve opening (taking the pressure delay into account and activating the DEA this delay earlier). Figure 3b shows the concept of the first protocol highlighting three different phase shifts (0%, 45%, and 90%). Fifteen different phase shifts were performed from 0% to 90%, with steps of 5% from 0% to 50%, and step of 10% from 50% to 90% (except for Animal 4, in which the protocol was performed with steps of 10% only, and thereby for 10 different phase shifts). In the second protocol (Protocol 2), the expected best phase shift with actuation mainly during systole was used with the actuation start and end times synchronized to the aortic valve opening and closure, respectively (taking the pressure delay into account and activating/deactivation the DEA this delay earlier). The start and end times of activation were shifted with −5%, 0% and 5% (of valve cycle) compared to valve opening and closure, respectively, giving a total of nine different recordings (three start times and three end times). Figure 3c shows the concept of the second protocol highlighting the three different start and end times. The tables in Figure 3b,c give the full overview of the two protocols with 15 and 9 different phase shifts and start and end times, respectively.

### 4.4 Hardware equipment

The complete hardware setup to convey the experimentation was separated in two parts: activation of the DEA and data acquisition (Figure 2c). A CompactDAQ (National Instruments, Austin, USA) connected to a computer with LabVIEW (National Instruments) was used to output the activation signal to the high‐voltage amplifier (Trek 20/20C; Advanced Energy, Denver, USA). The amplifier was connected to the DEA through wires and a small insulating box that allowed to secure the pig and medical staff from the high voltage. The pacemaker had two identical outputs: one for the pacing of the heart and the other for triggering the DEA activation. The activation profile was defined in LabVIEW as shown in Figure S13 by the period of the signal, the rising and falling time, the size of the plateau, the delay for the activation compared to the pacemaker signal, the voltage value, and the number of cycles. The rising and falling times (50 ms) were reduced as much as possible to be closer to a square signal while limiting the current flowing in the DEA that could lead to deterioration of the performances. As the pacing was very repeatable, the pacemaker triggered the activation only once at the beginning and the synchronization was ensured by the identical value between pacemaker pacing period and defined period in the program. To ensure, the signals were not shifted due to electromagnetic compatibility issues, the trigger signal from the pacemaker was galvanically isolated through a magnetic transformer. In Figure S14, we can see the superposition of all the activation signals compared to the pacing signal. We can see a small drift of maximum 3 ms between the 60 activations, mainly due to a slight error between the pacing period and the activation period that we considered not significant in the analysis of data.

All the signals from the experiment were acquired through two Powerlabs (ADInstruments) of 16 and 8 input ports. The latter one was used to obtain the signals from the pressure–volume catheter while the former one acquired the signals from the pressure catheters, the flow probes as well as the pacemaker trigger signal and the current and voltage monitoring values from the high‐voltage amplifier. A computer with LabChart Pro (ADInstruments) was used to visualize the signals in real time and save the data at a sampling frequency of 1 kHz.

### 4.5 Statistics

Pressure, flow, and volume were recorded continuously using LabChart. All protocols were performed for 60 consecutive heart cycles with actuated DEA, with at least 60 consecutive heart cycles baseline before and after the actuated cycles. Data from the two (*n* = 2) last animals were used in the analysis, due to different problems occurring during the three first experiments (e.g., early electrical breakdown of the device or early death of the animal). This study followed the 3Rs (Replace, Reduce, Refine) principles^27^ in which researchers are obliged to keep animal experiments to a minimum. Because this was an in vivo feasibility study (to test whether our DEAs could support the cardiac system) the low number of animals was justified and followed the 3Rs principle. Table S1 gives an overview of all five animals and all DEAs and protocols performed. No useful data could be recorded in Animals 1 and 3, due to early electrical breakdown of the devices and early death of the animal, respectively. In Animal 2, instable hemodynamic parameters during the recordings made analysis of the results challenging, and they were therefore excluded from the reported analysis. Figure S7A,B shows the results for Protocol 1 and 2, respectively, in Animal 2 with DEA actuation at 6.5 kV for two consecutive heart cycles compared to baseline. In Animals 4 and 5, all the 180 consecutive heart cycles for each protocol and each DEA were exported to MATLAB (Mathworks, Natick, MA, USA) for further analysis. For each protocol and DEA, only 20 heart cycles of baseline and 20 actuated heart cycles were picked and used in the analysis. The 40 heart cycles were consecutive, either using the baseline before actuation and the first cycles of actuation or using the last part of actuation and first part of baseline after actuation. For each cycle, the maximum, minimum, and mean values of all the pressure, flow, and volume measurements were calculated. The following parameters were calculated for all analyzed heart cycles: average left ventricular pressure (avg. LVP), end‐diastolic pressure (EDP), early diastolic aortic pressure (AoP dia, calculated as the average aortic pressure after valve closure until 0.1 s before aortic valve opening to exclude the last part of diastole), and average aortic flow (avg. Qao). Averaged heart cycles for all pressure and flow measurements were also calculated. The actuated heart beats were compared to the baseline measurements for all parameters, and the average values as well as standard deviations of all parameters were calculated. For each protocol (all 15 phase shifts and all 9 start and end times), the Wilcoxon signed‐rank test (*signrank* in MATLAB) was performed to compare the actuated heart cycles with the baseline cycles. A *p* value below 0.05 was considered significant.

## AUTHOR CONTRIBUTIONS

**Thomas Martinez:** Conceptualization (equal); data curation (equal); formal analysis (equal); investigation (equal); methodology (equal); software (equal); visualization (equal); writing – original draft (equal); writing – review and editing (equal). **Silje Ekroll Jahren:** Data curation (equal); formal analysis (equal); investigation (equal); methodology (equal); software (equal); visualization (equal); writing – original draft (equal); writing – review and editing (equal). **Armando Walter:** Data curation (equal); investigation (equal); methodology (equal); writing – original draft (equal); writing – review and editing (equal). **Jonathan Chavanne:** Conceptualization (equal); data curation (equal); investigation (equal); methodology (equal); software (equal); writing – review and editing (equal). **Francesco Clavica:** Data curation (equal); formal analysis (equal); investigation (equal); methodology (equal); writing – original draft (equal); writing – review and editing (equal). **Lorenzo Ferrari:** Data curation (equal); formal analysis (equal); investigation (equal); methodology (equal); writing – review and editing (equal). **Paul Philipp Heinisch:** Conceptualization (equal); investigation (equal); writing – review and editing (equal). **Daniela Casoni:** Investigation (equal); resources (equal); writing – review and editing (equal). **Andreas Haeberlin:** Formal analysis (supporting); investigation (supporting); writing – review and editing (equal). **Markus M. Luedi:** Investigation (supporting); writing – review and editing (equal). **Dominik Obrist:** Formal analysis (supporting); supervision (equal); writing – review and editing (equal). **Thierry Carrel:** Conceptualization (equal); funding acquisition (equal); writing – review and editing (equal). **Yoan Civet:** Conceptualization (equal); funding acquisition (equal); investigation (equal); methodology (equal); project administration (equal); supervision (equal); writing – original draft (equal); writing – review and editing (equal). **Yves Perriard:** Conceptualization (equal); funding acquisition (equal); project administration (equal); supervision (equal); writing – review and editing (equal).

## Supporting information


**Video S1** Working principle of the dielectric elastomer augmented aorta

**Fig. S1** Structure of the multilayered DEA
**Figure S2**. DEA fabrication steps
**Figure S3**. Close‐up of the housing connectors
**Figure S4**. Testbench for in vitro pressure volume characterization of the DEA
**Figure S5**. Pressure–volume Characteristics of the two DEA designs used during the in vivo experiment
**Figure S6**. Energy supplied by the actuator during protocol 1
**Figure S7**. Demonstration of the cardiac assistance from the DEA augmented aorta in Animal 2.
**Figure S8**. Sensor delay and calibration setup
**Figure S9**. Delay between DEA activation and aortic valve opening
**Figure S10**. Box‐plots of the variation of pressure and flow parameters
**Figure S11**. Pressure and flow signals for the full duration of protocol 1 in Animal 4
**Figure S12**. Chaotic behavior of the left ventricle volume measurement
**Figure S13.** Parameters defining the activation signal
**Figure S14**. Shift between pacing and activation signal
**Table S1**. Overview of all animals, DEAs tested, and protocols performed.
**Table S2**. Average values and standard deviations for all measured parameters

## Data Availability

The data that support the findings of this study are available from the corresponding author upon reasonable request.
