# Supplementary material for: A novel soft cardiac assist device based on a dielectric elastomer augmented aorta: An in vivo study
Source: Bioeng Transl Med. 2022 Aug 22;8(2):e10396. doi: 10.1002/btm2.10396 (PMC10013878; doi:10.1002/btm2.10396)
Supplement: Supplementary file 2 — Fig. S1 Structure of the multilayered DEA Figure S2. DEA fabrication steps Figure S3. Close‐up of the housing connectors Figure S4. Testbench for in vitro pressure volume characterization of the DEA Figure S5. Pressure–volume Characteristics of the two DEA designs used during the in vivo experiment Figure S6. Energy supplied by the actuator during protocol 1 Figure S7. Demonstration of the cardiac assistance from the DEA augmented aorta in Animal 2. Figure S8. Sensor delay and calibration setup Figure S9. Delay between DEA activation and aortic valve opening Figure S10. Box‐plots of the variation of pressure and flow parameters Figure S11. Pressure and flow signals for the full duration of protocol 1 in Animal 4 Figure S12. Chaotic behavior of the left ventricle volume measurement Figure S13. Parameters defining the activation signal Figure S14. Shift between pacing and activation signal Table S1. Overview of all animals, DEAs tested, and protocols performed. Table S2. Average values and standard deviations for all measured parameters [file BTM2-8-e10396-s002.docx]

**Supplementary Materials for**

A novel soft cardiac assist device based on a dielectric elastomer augmented aorta: an *in vivo* study

**Authors:** Thomas Martinez*, Silje Ekroll Jahren, Armando Walter, Jonathan Chavanne, Francesco Clavica, Lorenzo Ferrari, Paul Philipp Heinisch, Daniela Casoni, Andreas Haeberlin, Markus M Luedi, Dominik Obrist, Thierry Carrel, Yoan Civet, Yves Perriard

*Corresponding author. Email: thomas.martinez@epfl.ch

**The file includes :**

Materials and Methods

Fig. S1. Structure of the multilayered DEA

Fig. S2. DEA fabrication steps

Fig. S3. Close-up of the housing connectors

Fig. S4. Testbench for *in vitro* pressure volume characterization of the DEA

Fig. S5. Pressure-Volume Characteristics of the two DEA designs used during the *in vivo* experiment

Fig. S6. Energy supplied by the actuator during protocol 1

Fig. S7. Demonstration of the cardiac assistance from the DEA augmented aorta in Animal 2.

Fig. S8. Sensor delay and calibration setup

Fig. S9. Delay between DEA activation and aortic valve opening

Fig. S10. Box-plots of the variation of pressure and flow parameters

Fig. S11. Pressure and flow signals for the full duration of protocol 1 in Animal 4

Fig. S12. Chaotic behaviour of the left ventricle volume measurement

Fig. S13. Parameters defining the activation signal

Fig. S14. Shift between pacing and activation signal

Table S1. Overview of all animals, DEAs tested, and protocols performed.

Table S2. Average values and standard deviations for all measured parameters

Video S1. Working principle of the dielectric elastomer augmented aorta

**MATERIALS AND METHODS**

**Fabrication of the DEA**

The tubular actuator is composed of several layers of modules. Each of them contains a silicone film with its support, a silver track and a carbon electrode (Fig. S1A)). The stacking is done in such a way that two negative electrodes encapsulate the positive electrode (Fig. S1B). The module is produced using an elastomeric film (ELASTOSIL® 2030, Wacker, Germany) as a substrate. The first step is to prepare it with a CO_2_ laser cutter (TROTEC 360 flexx, Austria). It includes deposition zones and alignment marks engraving, making holes for the wire access, and cutting the module at the right size. 23 µm thick PET masks for screen-printing are also prepared at this time. Once the module is ready, a silver trace (125-19(SP) A/B Creative Materials) is screen-printed on the silicone surface (ZAA 2300 Zehntner, Proceq, Switzerland) (Fig. S2A), and cured in the oven at 80 °C. The purpose of this track, which is flexible and stretchable, is to electrically access the electrode, to reduce the access resistance and to distribute the charges. The electrode is then also screen-printed but using a homemade ink based on carbon powder (KETJENBLACK EC-600JD, Ströhle Chemicals, Switzerland), silicone (LSR-4305, Elkem, France), Isopropanol and Isooctane (Fig. S2B). The dimensions of the carbon electrode are defined using a PET mask and the curing is done in the oven at 80 °C for 4 hours. For one actuator three modules and one blank elastomeric film are necessary. The thickness of the silicone sheet depends on the location of the module in the stack. The external module, and the blank film, which serve as insulation, are 20 μm thick and the active layers are 100 μm thick (Fig. S1B). The modules are then stacked and fixed with a mixture of silicone (LSR-4305, Elkem, France) and carbon (KETJENBLACK EC-600JD, Ströhle Chemicals, Switzerland). The purpose of adding this powder is to make the silicone slightly less resistive, thus smoothing the electric field and avoiding a concentration that would lead to breakdown. When overlaying, it is important to ensure that the silver traces and holes are aligned. Otherwise, it will not be possible to wire the electrodes. Figure S1C shows this alignment and the position that the tracks must have to be accessed. Each track is staggered.


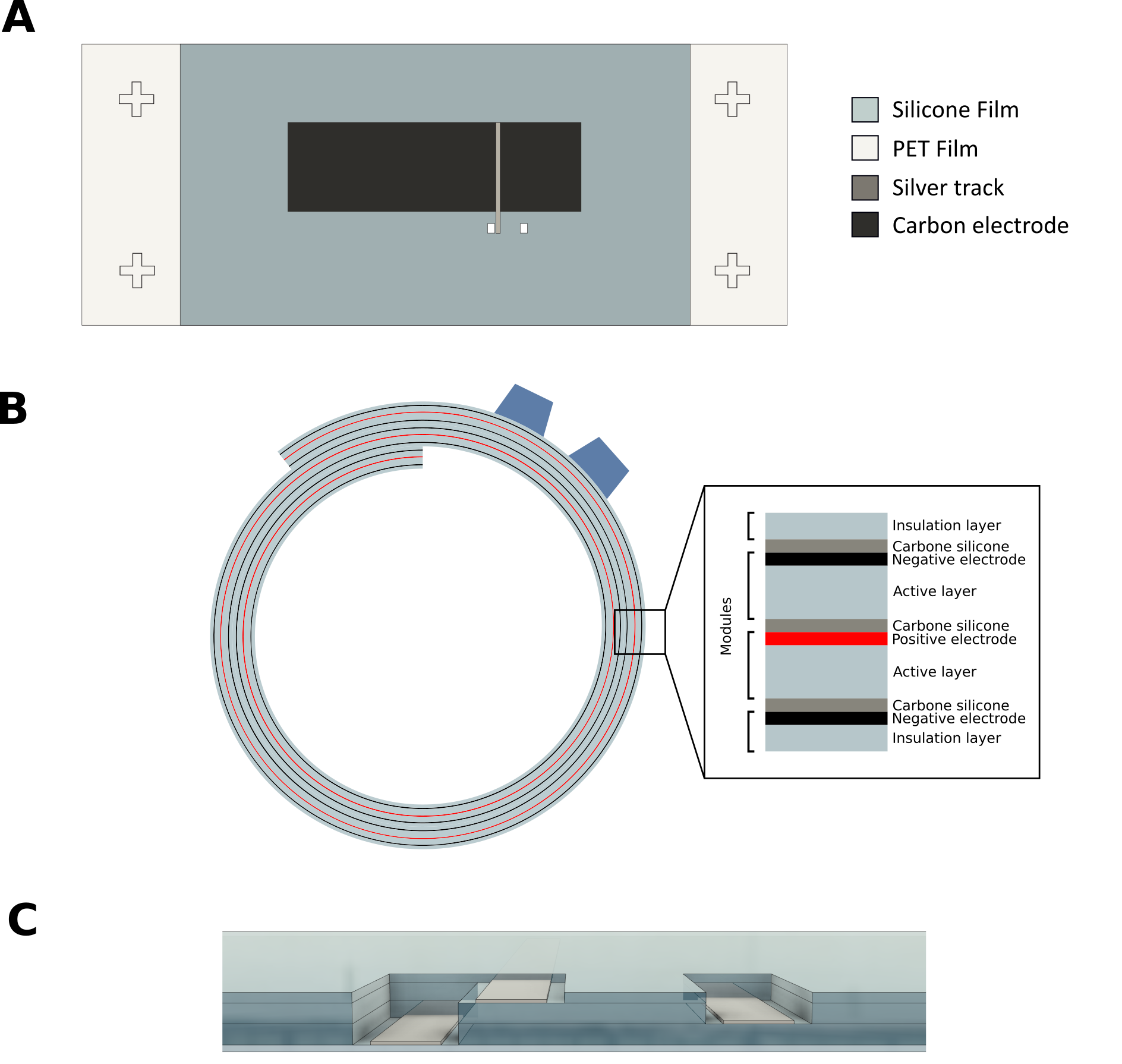


**Fig. S1. Structure of the multilayered DEA. (A)** One module is composed of silicone film (in light blue) on its PET film support (in white), alignment crosses engraved with a laser, silver track (in grey) and carbon electrode (in black). **(B)** Section view of the multilayered structure of the DEA after rolling with zoom on the stacking of modules. Two negative electrodes encapsulate the positive electrode. The two outer elastomer films are thinner (20 μm) and serve as insulation, while the two inner films, called active layers, are thicker (100 μm). Between each module, a film of carbon silicone is coated to fix them. For simplicity, silver ink is not showed here. **(C)**  Arrangement of the silver tracks for the connection with the electrodes

After stacking the four modules, a thin film of silicone (LSR-4305, Elkem, France) is coated and the DEAs are rolled around a 20 mm diameter PMMA (Poly(methyl methacrylate)) tube (Fig. S2C). A few hours in the oven at 80 °C allows the silicone to cure and maintain the DEA in the form of a cylinder. Then, two wires are added and fixed with a conductive silicone (ELASTOSIL® LR3162 A/B, Wacker, Germany) on each hole. To insulate and be able to put the device in a humid environment, silicone (Sylgard 186, Dow Corning, USA) is casted on top of each electrical connectors (Fig. S2D). It is very important to consider the material of the wire sheath and make sure that it bonds well with the silicone insulation. If it doesn't, liquid can seep in and create a short circuit. To avoid this, the electric wire can be inserted in a silicone duct.


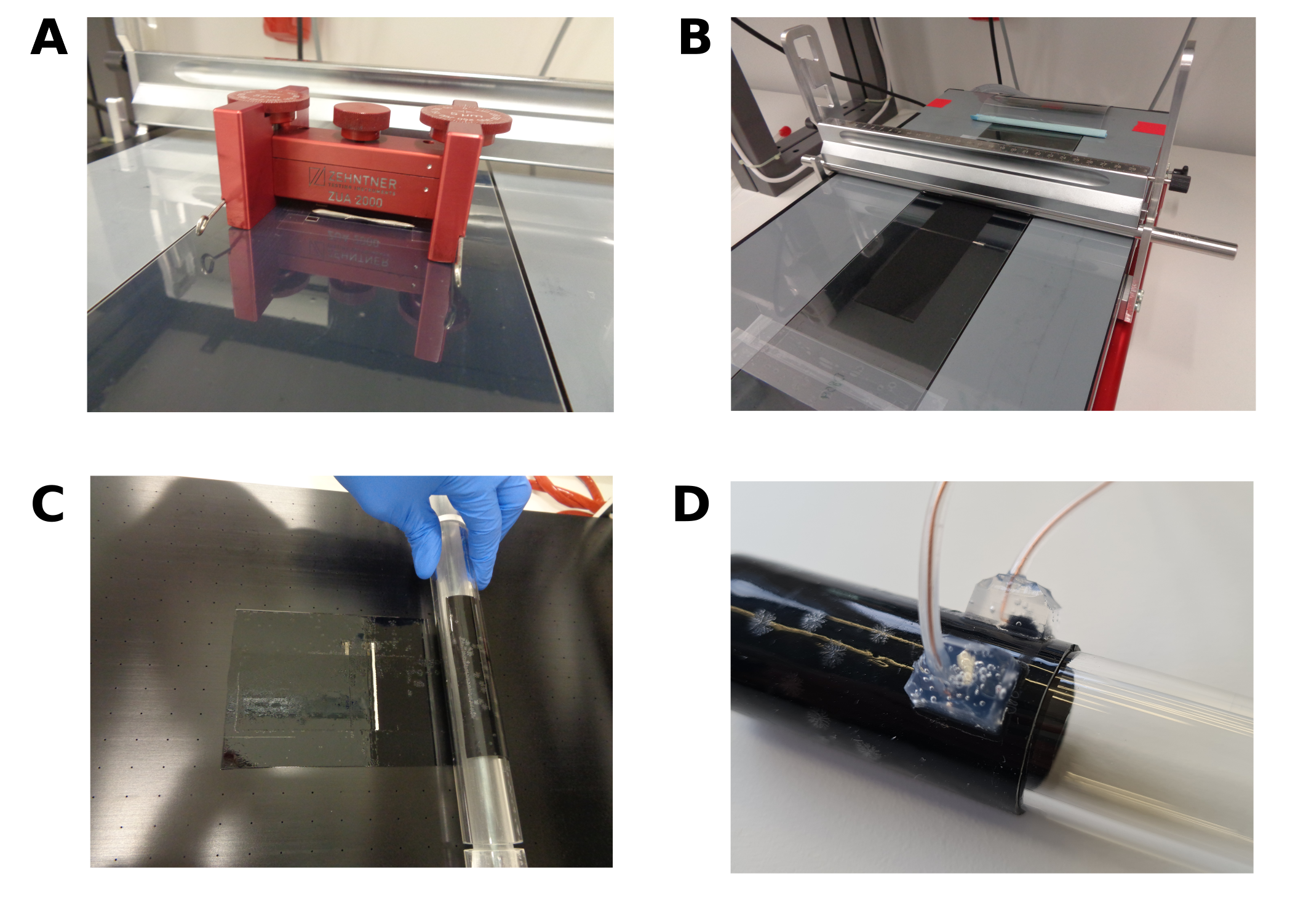


**Fig. S2. DEA fabrication steps. (A)** Deposition of the silver ink line on top of the Elastosil film. **(B)**Deposition of the carbon electrode on top of the Elastosil film through screen printing. Two passages are done to ensure regular deposition over the surface of the film. **(C)**Rolling of the stack of modules over a PMMA tube to obtain the final DEA. **(D)**Electric insulation of the DEA electrical connections. A wire with a silicone hollow tube around is connected to the silver lines with carbon-doped silicone. A coating of silicone around the electrical connections and the wire ensures electrical insulation and mechanical attachment

A housing has been designed and 3D printed to enclose the DEA. The main goals of this housing are to protect the rolled DEA from the external compression and from folding, to limit the expansion of the device and to help the surgeon connect it to the aorta. It is composed of four main parts: two connectors and two half cases. Figure 1E shows these different parts of the implant. The small holes covering the case are designed to facilitate the entry and exit of fluids.


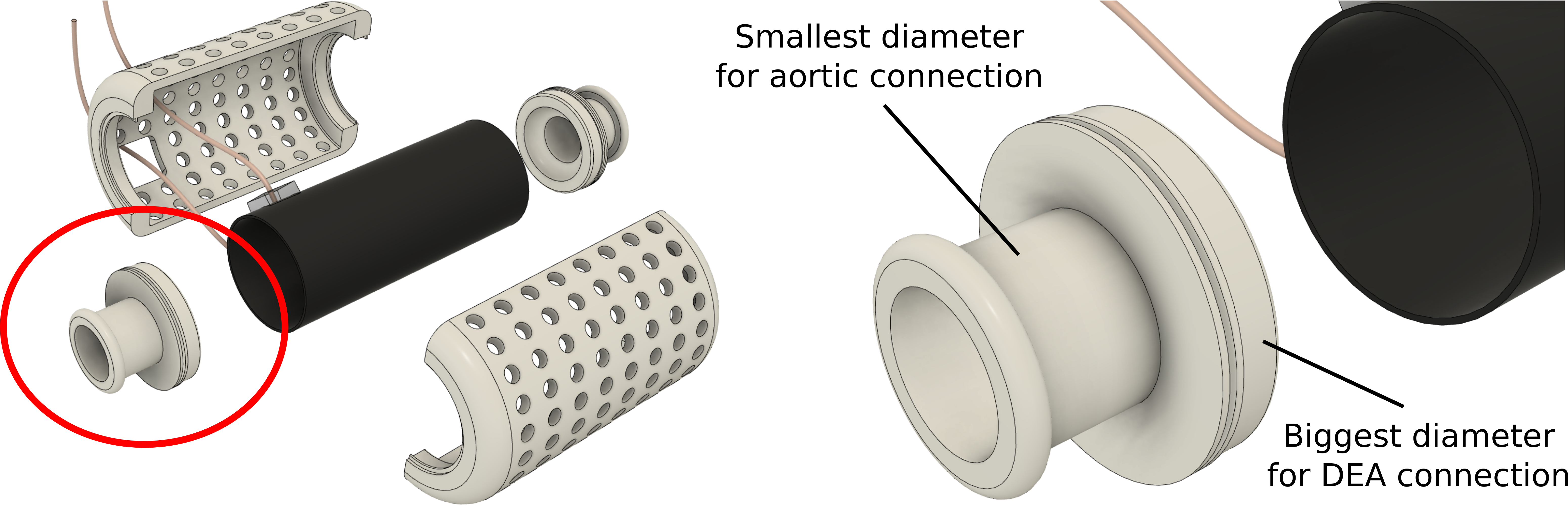


**Fig. S3.** **Close-up of the housing connectors.** The biggest diameter section conforms to the DEA diameter to ensure sealing of the connection between the DEA and the external housing. The smallest diameter section is inserted in the aorta lumen and fixed to the aorta with plastic clamp ties. The diameter step at the end of this section ensures the good sealing of the anastomosis.

The dimensions of the DEA were chosen accordingly to the space available in the descending aorta as well as the pressure levels inside the pig. The diameter and length of the DEA need to be maximized to obtain the highest volume displacement and thus the highest influence of the device. On the other hand, the overall thickness of the device must be designed more finely. If the DEA is too rigid, it will not deform enough to influence the hemodynamic parameters. On the other hand, a too compliant device would highly deform even when passive. In this case, the DEA can break very early as its thickness is already low. Finally, with the fabrication process presented earlier, the final number of active layers in the rolled DEA must be at minimum 4. Moreover, the thickness of the sheets are also limited and the final thickness of the DEA cannot be designed very accurately and must take into account these parameters. The final DEA was chosen to be as around 500 µm overall thickness to fit the most to the pressure levels inside the pig.

**Pressure Volume Characterization of the DEAs and energy determination**

Characterization of the DEA was performed before *in vivo* experiments. The aim of this experiment was to obtain the pressure volume characteristics of 29 mm and 39 mm DEAs. The device consists of the DEA and its enclosure, and two rigid supports were fixed on each side as seen in Fig. S4A. On one side, a pressure sensor (Baumer PBMN-25B12, Frauenfeld, Switzerland) was connected while on the other extremity, the whole device was connected to a pneumatic system composed of a piston, a motor and a dead volume of air through an inlet pressure (Fig. S4B). All this assembly was mounted on a mandrel. The control of the motor displacement determines the volume of air going inside the DEA and the resulting pressure was measured by the sensor at the bottom. The dead volume ensures that when activated the pressure does not drop drastically. The DEA was connected to the 20/20 high voltage amplifier from TREK. Finally, a 2D laser measured the deformation profile along the length of the actuator through a slot in the enclosure from which the volume was determined by assuming axisymmetric deformation. The control of the motor displacement, the activation of the DEA and the acquisition of the data except the laser was done in LabVIEW through a DAQ (NI USB-6259, National Instruments, Austin, USA) at 50 kHz sampling frequency. The laser data were extracted after a test and had a sampling frequency of 100 Hz. All the data were then processed and synchronized in MATLAB.


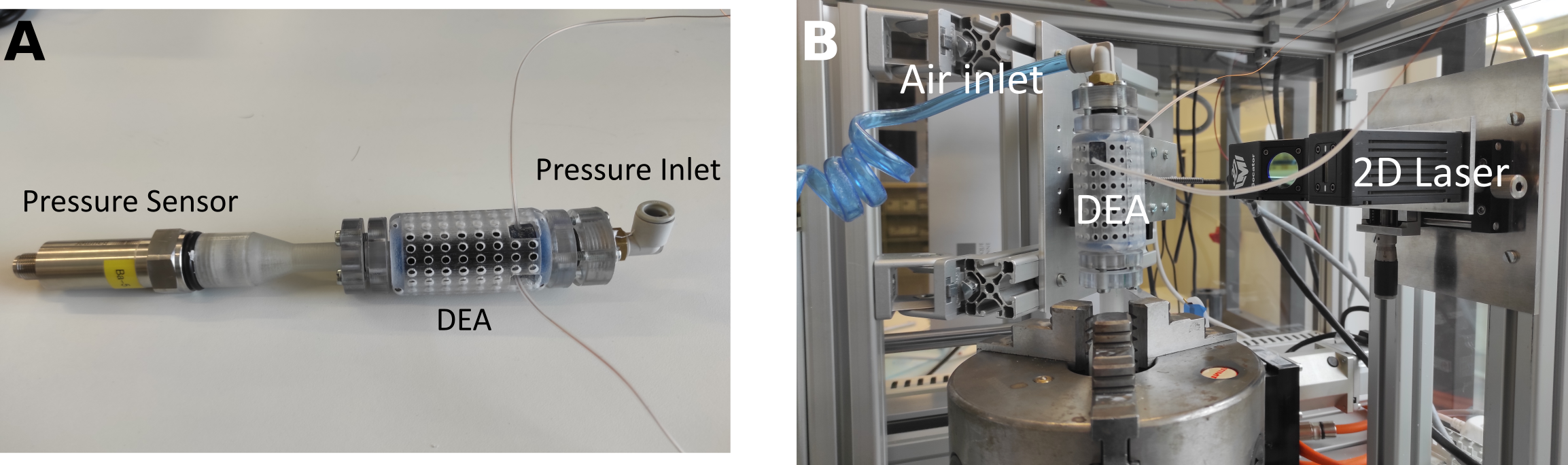


**Fig. S4. Testbench for in vitro pressure volume characterization of the DEA** **(A)** Support of the DEA for testing on the Pressure-Volume testbench. **(B)** Full testbench for the pressure-volume measurement. The DEA support is mounted on a mandrel with the pressure sensor hidden here in it. The motor pushes air inside the DEA through the pressure inlet. The 2D sensor measures the displacement profile of the DEA over the length of the tube to obtain the volume deformation of the actuator.

A device was first tested at 0 kV. We increased the pressure inside the DEA by moving the piston until it reached 90 mmHg then the piston went back to its initial state. The 2D profile was measured in the meantime and the data were then put together to obtain the pressure-volume characteristics. The device was then tested in the same way but with increasingly higher voltages from 0.5 kV to 7 kV with 500 V steps. The only difference was that the pressure was first increased in the DEA to 30 mmHg and only then the DEA was activated. This ensured that the DEA was not wrinkled when the voltage increased. The final pressure volume characteristics are shown in Fig. S5.


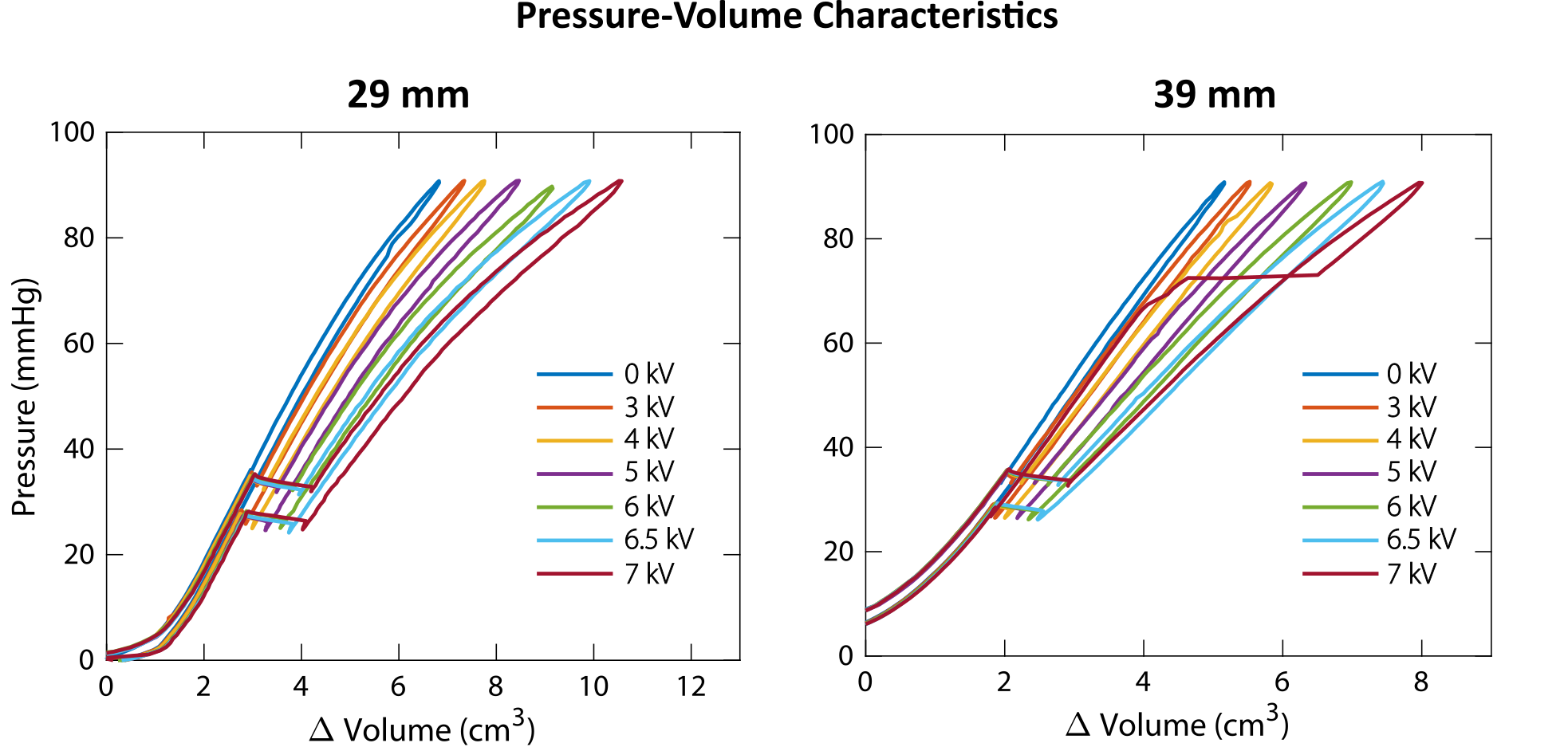


**Fig. S5. Pressure-Volume Characteristics of the two DEA designs used during the in vivo experiment.** Both DEAs were tested up to 90 mmHg and 7 kV. The hysteretic behavior is due to the viscoelastic properties of the elastomer, enhanced with increase voltage. For the 29 mm DEA, the specific behavior at 7 kV when the pressure went down is due to electric breakdown of the actuator but still allows to obtain the energy cycle for the DEA.

The protocol 2 results shown Fig. S6 do not allow to conclude on the best activation scheme from the DEA point of view. Indeed, as the activation cycles are very close, the energy differences between profiles are low. Moreover, the energy depends on the blood pressure level in the descending aorta and variations in this parameter during measurements of a full protocol leads to energy differences not linked specifically to the activation cycle of the DEA.


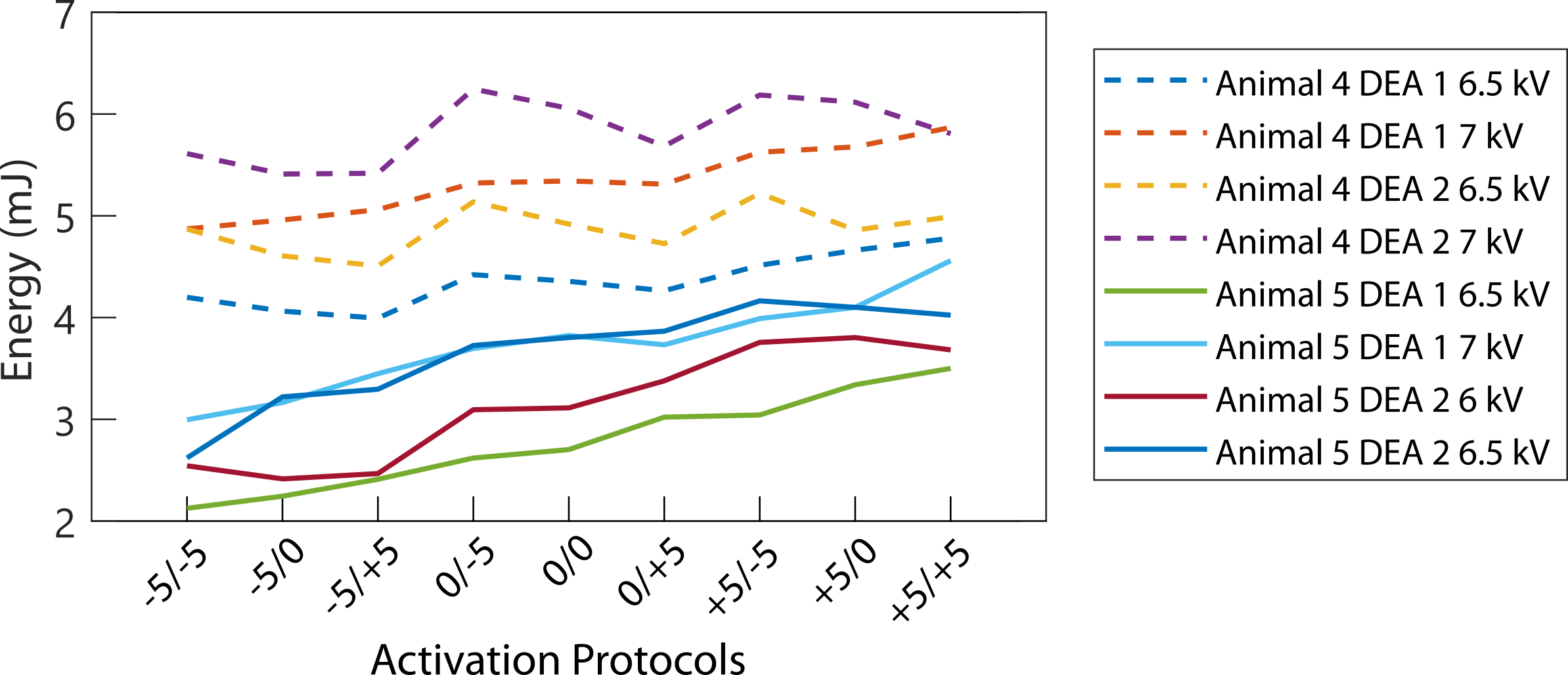


**Fig. S6. Energy supplied by the actuator during protocol 1.** We note an increase of the energy for late start of activation (+5,-) linked to later activation of the DEA and thus lower minimal pressure in the energy cycle. However, the differences of energy between different activation protocols are low and are hardly linkable to the evolution of physiological parameters observed.

**DEA cycling before *in vivo* experiments**

All the devices prepared for the *in vivo* experiments were submitted to preliminary tests. All the devices (tube + housing) were activated at 80 mmHg pressure with voltages going up to 6.5 kV to ensure the DEA would sustain these voltages when implanted in the pig. The device under test was connected to a water column through one of the two connectors. On the other end, a plug blocks the water flow. The level of water was set to obtain a pressure of 80 mmHg in the DEA when passive. The tube was then submitted to increasing voltages from 2 to 5 kV with 1 kV step. For these steps, 15 activations were performed. Then, 60 activations were done at 5.5 kV. Finally, the DEA was activated 10×60 times at 6 and 6.5 kV. The same complete process was then performed again with the tube fully immersed in water to ensure good insulation of the device especially near electric connections.

***In vivo* Experiment**

**Animals**

Table S1 shows the overview of the n = 5 pigs used in the *in vivo* experiments together with the DEAs tested and protocols performed. In the first three animals, different problems occurred. In animal 1, several DEAs experienced early electrical breakdown due to humidity, and no DEA protocols could be performed before the experiment was ended. In animal 2, the first two DEAs (DEA 8 and 9) broke down during the initial tests or during the first protocol, while for the third implanted DEA (DEA 10) we could perform both protocols for two different activation voltages. In this animal, the experimental protocol included repeated cycles of apnoea (ventilation of the animals turned off during recordings) to remove breathing artifacts and have stable hemodynamic parameters during the recordings. Unfortunately, this led to pressure and flow decrease during the measurements (hence instable hemodynamic parameters) and the quantification of the effect of the DEA became difficult. Figure S7A and B show the two last heart cycles before DEA actuation at 6.5 kV and the two first actuated heart cycles for protocol 1 and 2, respectively. The results are comparable to the reported results in animal 4 and 5 in the main manuscript. Animal 3 succumbed to a fatal arrhythmia during ventricular pacing before any tests could be performed. In the last two animals, we got good results and therefore these two animals were used in the analysis shown in the main manuscript.

**Table S1. Overview of all animals, tested DEAs, and performed protocols.**

**Fig. S7. Demonstration of the cardiac assistance from the DEA augmented aorta in Animal 2. (A)** Aortic pressure (upstream DEA), left ventricular pressure, DEA actuation voltage, and trigger (pacemaker signal) signals for two consecutive actuated heart cycles (ON) overlayed with two consecutive baseline cycles (OFF) for protocol 1 in animal 2 for ten different phase shifts with the DEA actuation with 6.5kV. **(B)** Aortic pressure (upstream DEA), left ventricular pressure, DEA actuation voltage, and trigger (pacemaker signal) signals for two consecutive actuated heart cycles (ON) overlayed with two consecutive baseline cycles (OFF) for protocol 2 in animal 2 for the nine different start and end times for DEA activation with the DEA actuation with 6.5kV. Trigger: pacemaker signal, PLV: pressure in the left ventricle, Pao: pressure in the aorta (upstream DEA), HighVolt: DEA actuation voltage.

**Anaesthesia**

Upon arrival at the testing facility, the animals went through a pre-anaesthetic clinical exam. Baseline recordings of heart rate, respiratory rate and temperature were performed. The animals were then sedated with intramuscular ketamine (10 mg/kg), dexmedetomidine (15 mcg/kg) and morphine (0.2 mg/kg). Further 3 mg/kg ketamine and 5 mcg/kg dexmedetomidine were injected in one pig as the sedation was insufficient after fifteen minutes. Under oxygen supplementation through a facial mask an intravenous cannula was placed in the marginal auricular vein and after preparation of the surgical field, the induction of general anaesthesia was achieved with ketamine (1 mg/kg) and propofol to effect (1-6 mg/kg). Amoxicillin and clavulanic acid (20 mg/kg) were administered after the induction of general anaesthesia. After intubation of the trachea, anaesthesia was deepened and maintained with sevoflurane in oxygen and compressed air. End tidal sevoflurane was adjusted to guarantee adequate depth of anaesthesia up to the MAC (2.7%). Additional analgesia was provided before thoracotomy with ropivacaine 0.75%, maximum of 2 mg/kg, and morphine 0.1 mg/kg injected in a spinal catheter introduced through median access at the lumbosacral space and advanced to the thoracic region targeting thoracolumbar junction for the sternotomy and T9-T10 for the intercostal access. Positive pressure ventilation was started after tracheal intubation using PEEP of 5cmH2O, a tidal volume of 8-12 mL/kg body weight, targeting a PaCO2 of 40-45 mmHg. A jugular four lumen catheter was inserted in the internal jugular vein after peri-incisional ropivacaine 2-4 mg/kg to record central venous pressure and an 8.5 French catheter in the external jugular vein for rapid volume infusion. Carotid and saphena arteries were catheterized to allow continuous measurement of arterial blood pressure. During general anaesthesia, heart rate, respiratory rate, arterial oxygen saturation, capnography, invasive blood pressure, esophageal temperature, inspired and expired fraction of gases (air, etCO2), central venous pressure and EEG were monitored through a multi-modular monitor (S/5 Critical Care Monitor®; Datex-Ohmeda, GE Healthcare, Helsinki, Finland). A mean arterial blood pressure (MAP) of 65 mmHg was targeted all over the anaesthesia and hypotension was addressed with the use of colloids, inotropes/vasopressors titrated to effect. To prevent arrhythmias induced by heart manipulations, amiodarone (3-5 mg/kg) was administered prior to the thoracotomy. Crystalloids and/or colloids were administered during the full course of anaesthesia. Heparin was administered and ACT was monitored routinely to target three times the baseline. A cardiac bypass was set in the animals 2-5 during aortic clamping. A forced air device (Mistral air) was used to maintain normothermia. At the end of the experiments, Pentobarbital 100 mg/kg was injected intravenously while the animals were unconscious due to general anaesthesia. EEG and ECG silence was used to confirm death. Animal 3 died spontaneously during ventricular pacing as fatal arrhythmia unresponsive to cardio-pulmonary resuscitation and defibrillation occurred.

**Correction of sensor delays and calibration of pressure sensors**

To remove all hardware and software induced sensor delays and thereby synchronize all sensors, we created a sensor delay and calibration setup. Figure S8 shows a schematic of the setup. All sensors were attached at the bottom of a water column at the same height. A falling cylinder created a sharp signal which was measured for all sensors. The time delay between the arrival of this sharp signal for the difference sensors compared to the arrival at a piezo element as reference were removed. Additionally, this setup was used to calibrate the pressure sensor by measuring three different reference heights of water.

**Fig. S8. Sensor delay and calibration setup.** All sensors are attached at the same height at the bottom of the water column. A falling cylinder generates a sharp pressure and flow signal measured by the DAQ and the measured sensor delays are removed. For the pressure sensor calibration three different static height of water were measured.

**Pressure delay in the descending aorta and wave propagation**

The DEA was positioned in the descending aorta in the *in vivo* experiments, and the pressure in the DEA was therefore delayed compared to the pressure in the left ventricle. Figure S9A shows the schematic of the wave propagation in the aorta and the definition of the delay. To synchronize the DEA with the left ventricle and give the waves generated by the DEA time to reach the left ventricle at wanted time points, this delay (t_delay_) was removed for the actuation of the DEA. Meaning the DEA was activated earlier with the same amount as t_delay_. Figure S9B shows the actuation voltage starting t_delay_ earlier than aortic valve opening, defined as actuation synchronized with aortic valve opening.


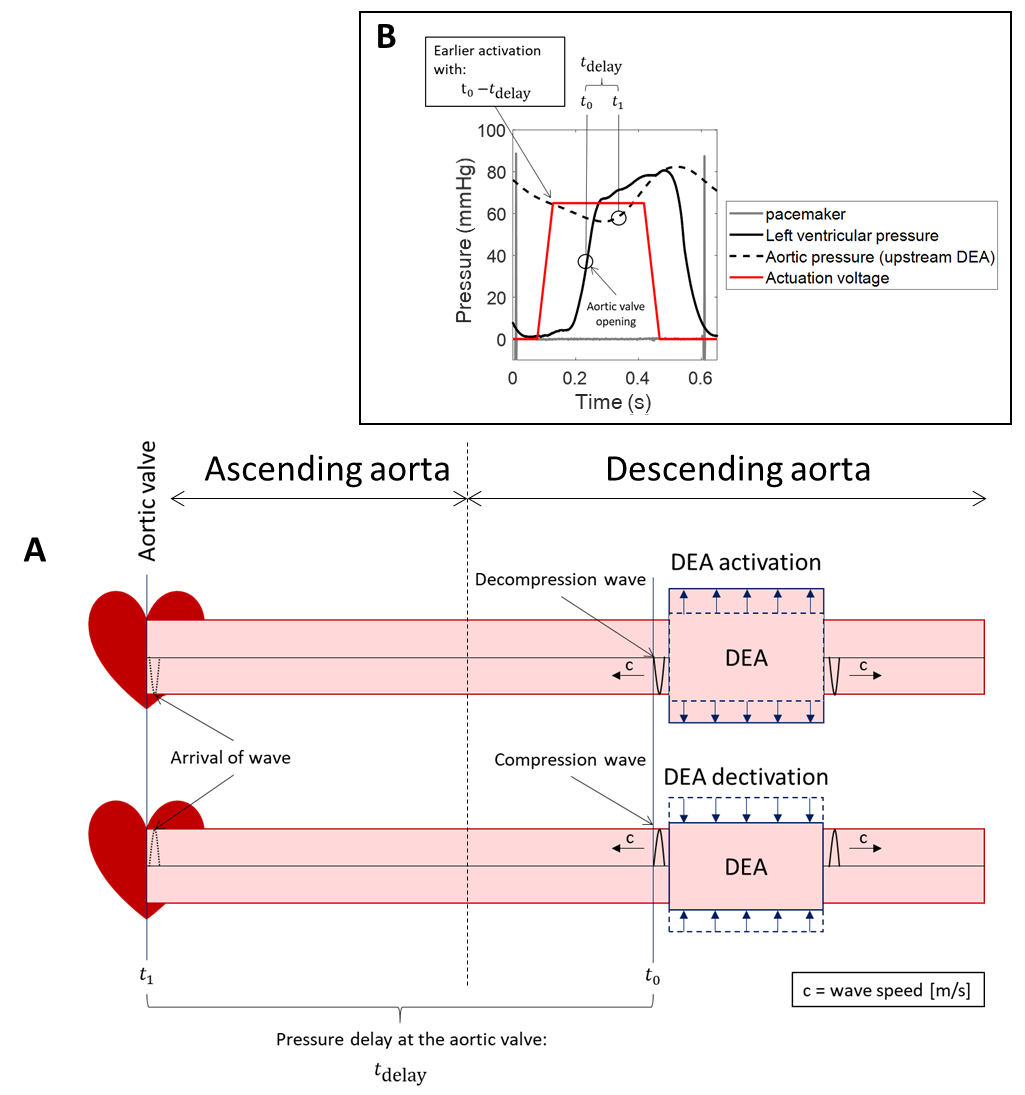


**Fig. S9. Delay between DEA activation and aortic valve opening. (A)** The DEA expands when activated and returns to its passive state when deactivated, and thereby creates a decompression wave and a compression wave, respectively, propagating upstream and downstream of the device. The propagating waves are travelling with the wave speed c and use the time t_delay_ to reach the aortic valve from the DEA positioned in the descending aorta. To synchronize the arrival of the DEA generated waves with the opening and closing of the aortic valve, the DEA is activated and deactivated with the time t_delay_ before aortic valve opening and closure, respectively. **(B)** Pressure in the left ventricle and the aorta upstream of the DEA, the pacemaker signal, and the actuation voltage. In this case the actuation of the DEA is started t_delay_ earlier than aortic valve opening to allow the decompression wave to reach the aortic valve at the time of aortic valve opening.

**Results**

Figure S10 shows the pressure and flow parameters for all phase shifts of protocol 1 (A) and all different start and end of activation of protocol 2 (B) aggregated for all analysed heart cycles compared to baseline. The same trends can be seen as for the average values for each DEA and voltage level in Fig. 4B and Fig. 6B in the main manuscript. Because the impact of the different DEAs on the parameters had a different amplitude, the trends are similar but less visible in the aggregated averages compared to the averages of each DEA separately. Table S2 gives an overview of the absolute value of all measured and calculated cardiac parameters in average and standard deviation for the analysed heart cycles in the main manuscript for protocol 1 (Table 1A) and 2 (Table 1B). Figure S11 shows the raw pressure and flow signals of the continuous measurement during protocol 1 in animal 4 with DEA 2 in which the largest effects were observed. Clearly, the aortic pressure upstream of the DEA (Fig. S11A), the LVP (Fig. S11B) and the aortic flow upstream of the DEA (Fig. S11C) reacts to the DEA actuation (Fig. S11D). Depending on the phase shift the amplitude of the effect is stronger and weaker, and leads to decrease or increase in the signals.


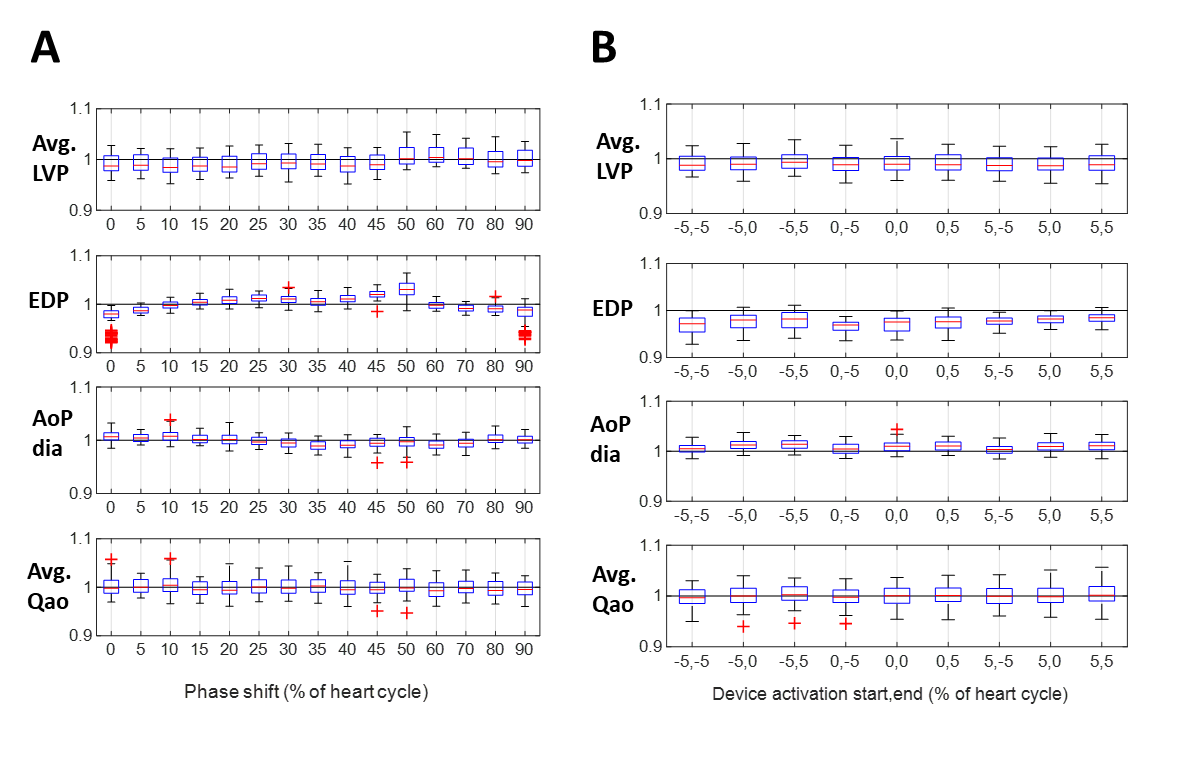


**Fig. S10. Box-plots of the variation of pressure and flow parameters.** The results are aggregated for all analysed heart cycles compared to baseline for protocol 1 (**A**) and 2 (**B**). Avg.: average, EDP: end-diastolic pressure, AoP dia: early diastolic aortic pressure, Qao: aortic flow upstream of DEA.


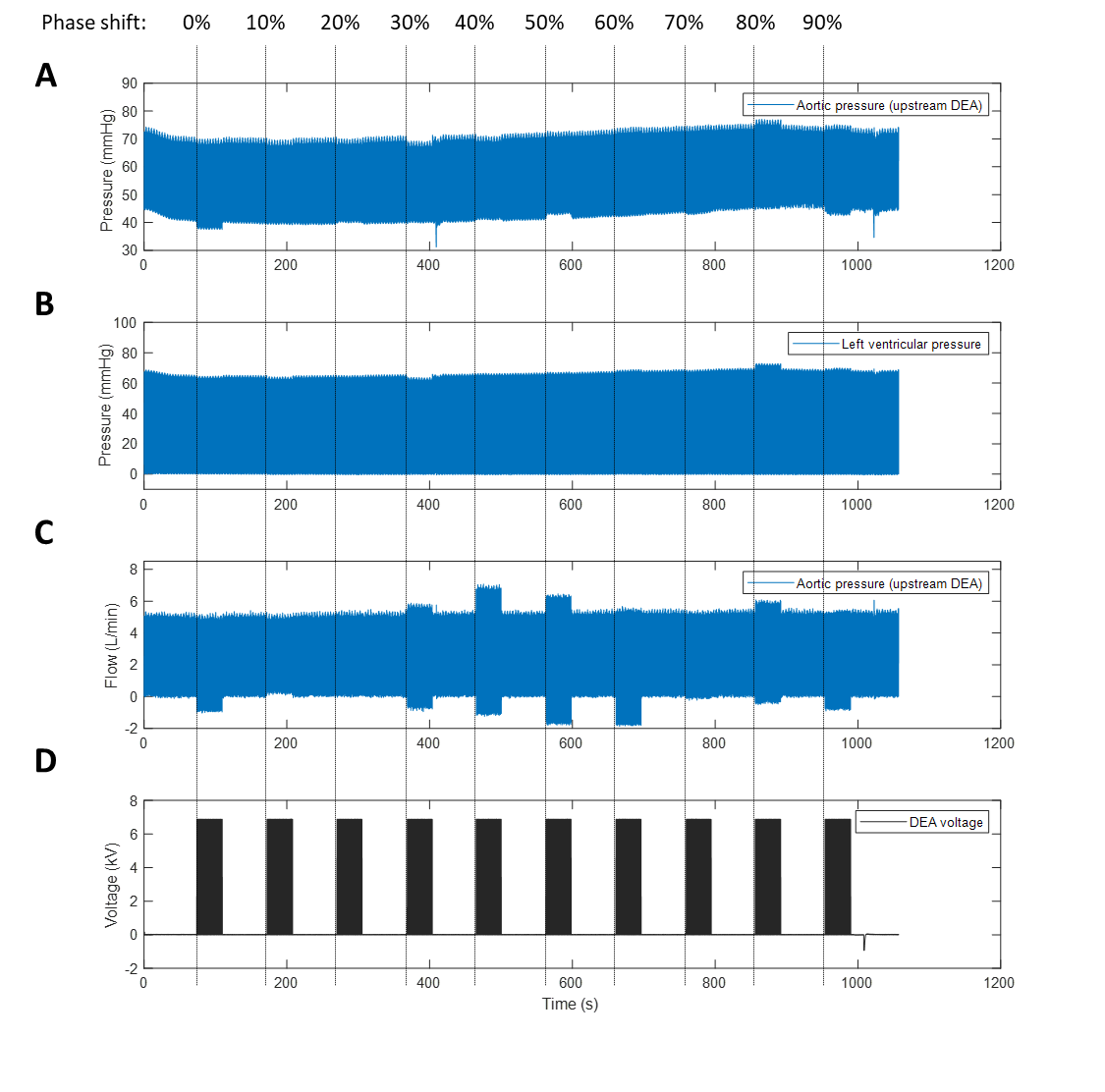


**Fig. S11. Pressure and flow signals for the full duration of protocol 1 in Animal 4. (A)** Aortic pressure upstream of the DEA. **(B)** Left ventricular pressure. **(C)** Aortic flow upstream of the DEA. **(D)** Actuation voltage. The vertical dotted lines mark the start of actuation for the 10 different phase shifts (0-90%) recorded.


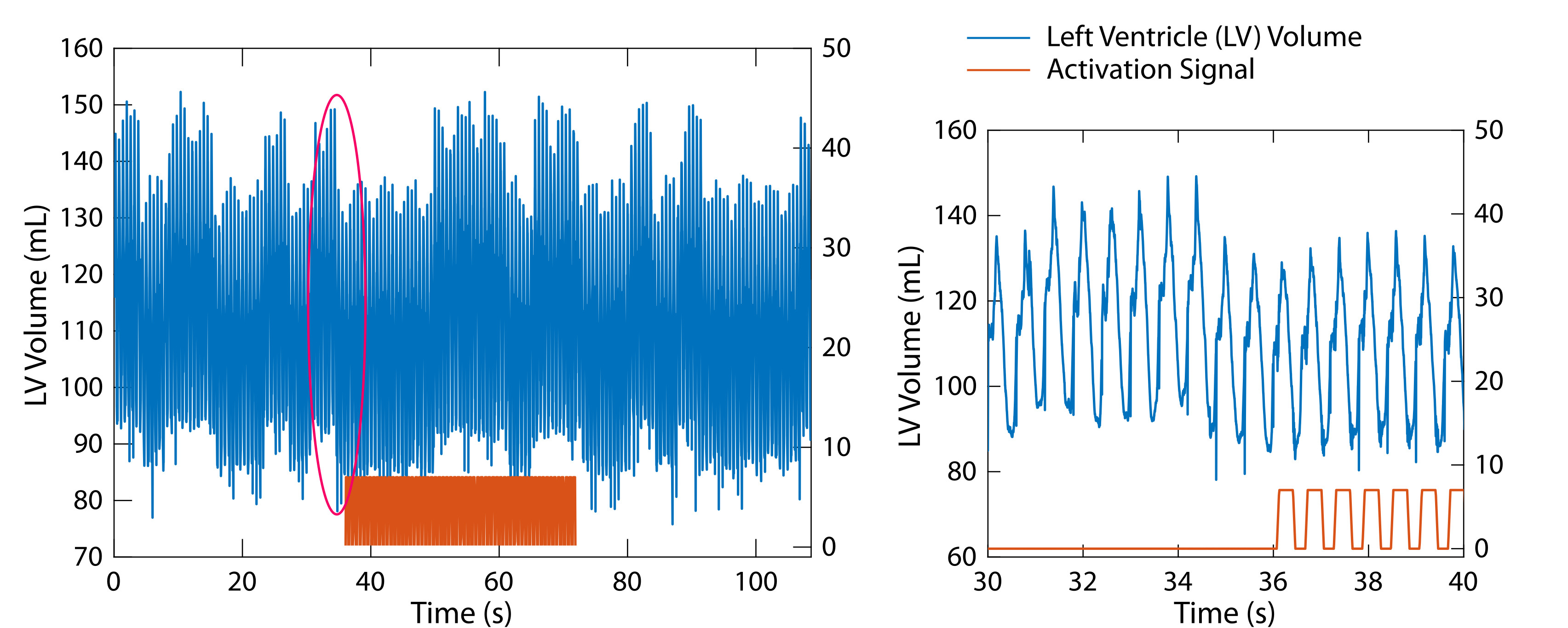


**Fig. S12. Chaotic behavior of the left ventricle volume measurement.** Measure of the left-ventricle volume as given by the pressure-volume catheter for animal 5, DEA 1, 7 kV, Protocol 2.1. Left: On the full range of time, we see the chaotic shift of volume up and down independent of the activation of the DEA. It seems also that this shift is not linked to the respiration of the animal as it does not follow a regular pattern. Right: a close-up on one of these shifts (red circle in left plot) near the start of the activation showing that the shift of volume is not linked to DEA activation.

**Table S2. Average values and standard deviations for all measured parameters.** The results are for all animals and DEAs used in the analysis given in absolute values for protocol 1 **(A)** and 2 **(B)**

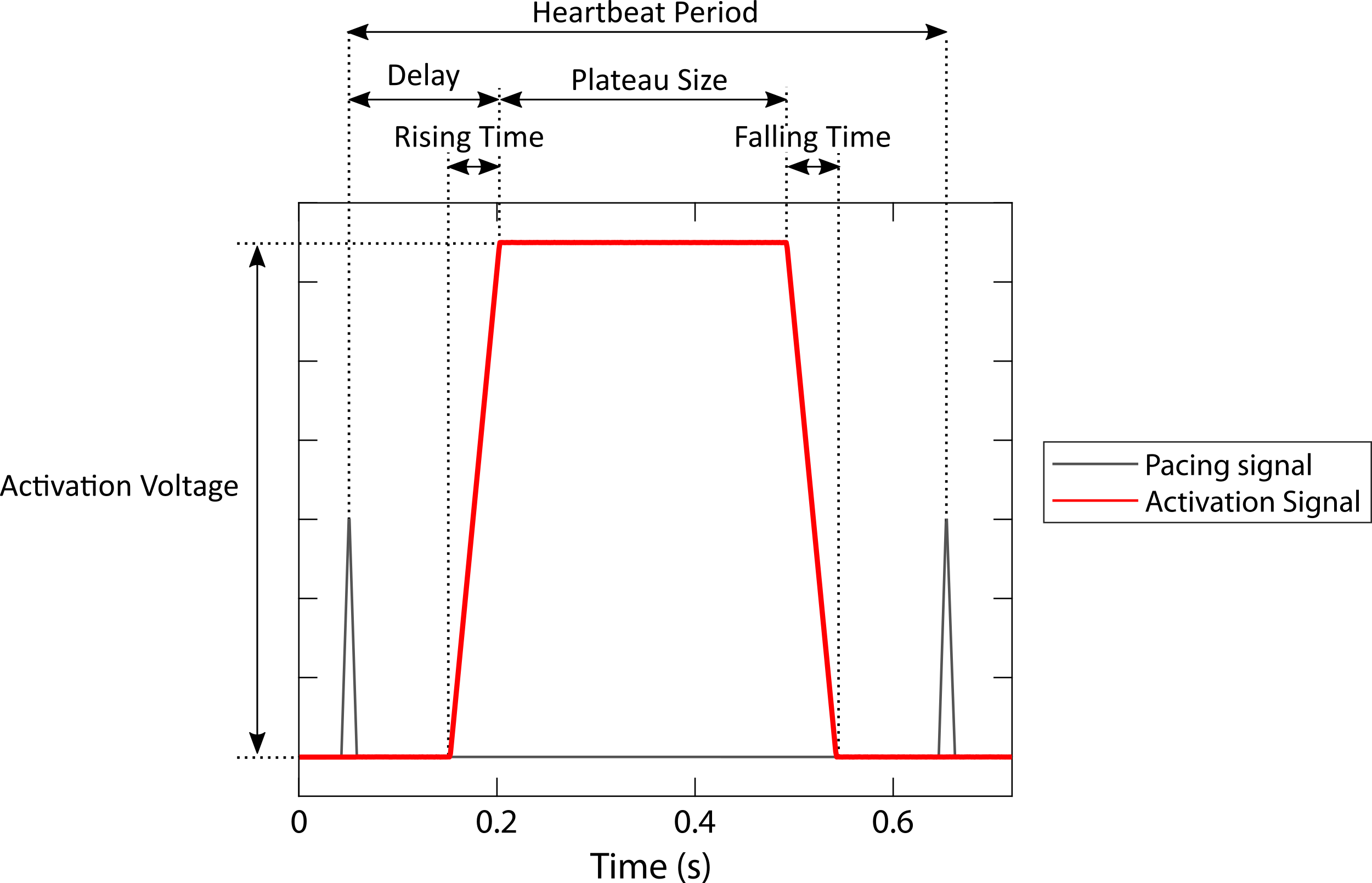


**Fig. S13. Parameters defining the activation signal.** The delay and plateau size are determined from delay measurement and aortic opening and closing timing as defined in Fig. 3A. The rising and falling times were reduced at maximum to limit


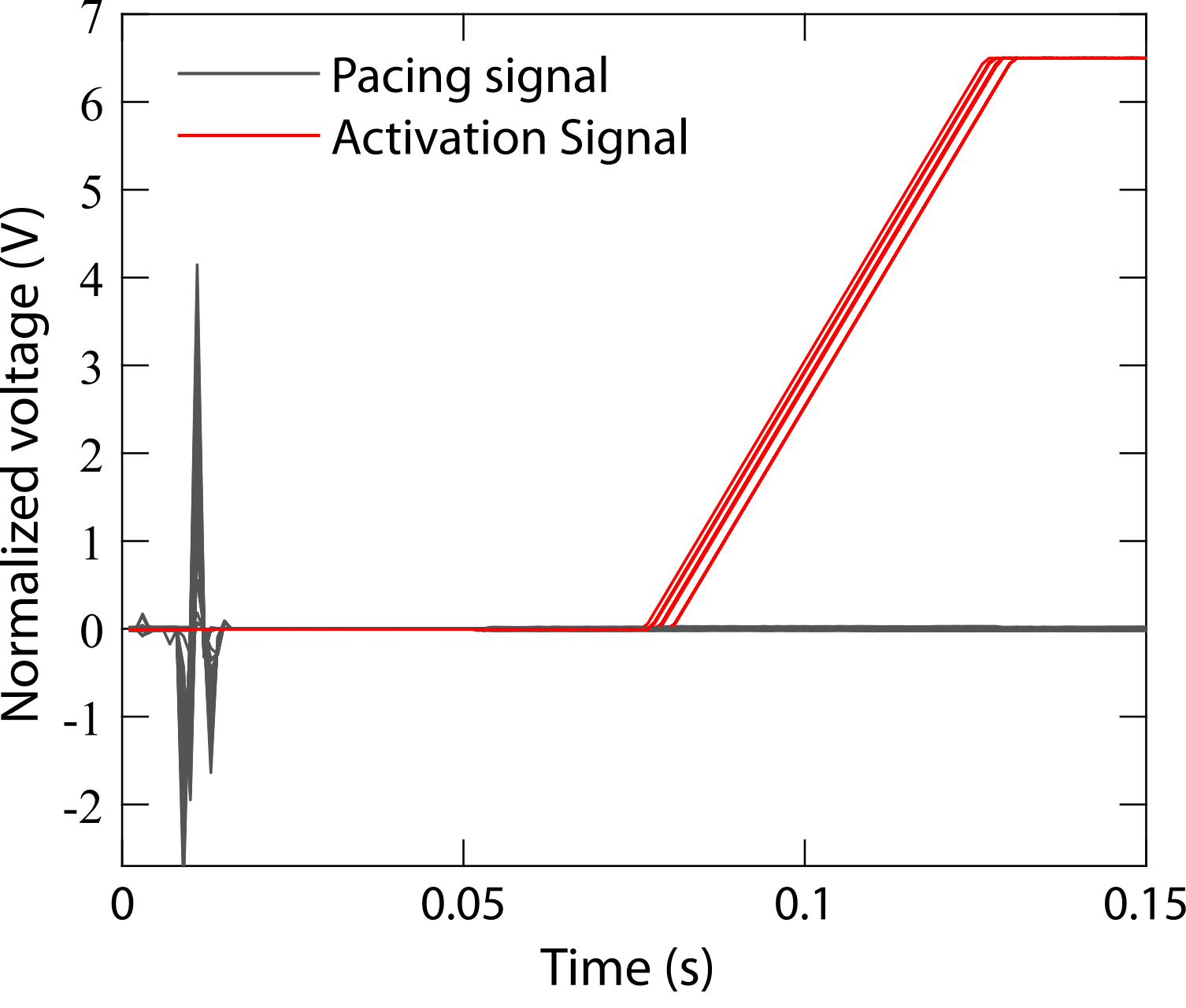


**Fig. S14. Shift between pacing and activation signal.** Superposition of pacing and activation signals for the 60 heartbeats during which the DEA is activated. All the pacing signals are represented at the same time point. The activation signal do not fully superposed as after 60 cycles a little shift might be induced from a slight error between the pacing period and the period defined for activation signal. As the sampling frequency is 1 kHz, we see a minimum of 1 ms shift between these signals. In the end, all the activation signals are in a 3 ms range.

**Video S1 : Working principle of the dielectric elastomer augmented aorta.** The start of the video shows the schematic implantation of the DEA as a replacement of the aorta. By activating the device in phase we the heart, the actuator can impact the haemodynamic parameters of the heart. The activation of the DEA during in vitro experiment is then superimposed to the schematic view to show how the DEA actually deforms.
